# The cerebellum converts input data into a hyper low-resolution granule cell code with spatial dimensions: a hypothesis

**DOI:** 10.1098/rsos.241665

**Published:** 2025-03-26

**Authors:** Mike Gilbert, Anders Rasmussen

**Affiliations:** ^1^ School of Psychology, University of Birmingham, Birmingham, UK; ^2^ Department of Experimental Medical Science, Lund University, Lund, Sweden

**Keywords:** cerebellum, theory, model, granular layer, network, code

## Abstract

We present a theory of the inner layer of the cerebellar cortex, the granular layer, where the main excitatory input to the cerebellum is received. We ask how input signals are converted into an internal code and what form that has. While there is a computational element, and the ideas are quantified with a computer simulation, the approach is primarily evidence-led and aimed at experimenters rather than the computational community. Network models are often simplified to provide a noiseless medium for sophisticated computations. We propose, with evidence, the reverse: physiology is highly adapted to provide a noiseless medium for straightforward computations. We find that input data are converted to a hyper low-resolution internal code. Information is coded in the joint activity of large cell groups and therefore has minimum spatial dimensions—the dimensions of a code group. The conversion exploits statistical effects of random sampling. Code group dimensions are an effect of topography, cell morphologies and granular layer architecture. The activity of a code group is the smallest unit of information but not the smallest unit of code—the same information is coded in any random sample of signals. Code in this form is unexpectedly wasteful—there is a huge sacrifice of resolution—but may be a solution to fundamental problems involved in the biological representation of information.

## 1. Introduction

It is generally assumed that brain-coded information is rich and replicable with computer-like high fidelity, and that those features make it precise and allow computations to be powerful. In any event, this is not regarded as contentious. In many neural network learning models, including cerebellar models, computations are implemented by learned synaptic changes that selectively adjust synaptic transmission strength. This has been a mainstay of much modelling for 50 years. We will argue, *inter alia*, that these influential ideas may need to be reconsidered.

The inner layer of the cerebellar cortex, the granular layer, receives the main excitatory input to the cerebellum, from mossy fibres. Mossy fibres contact both granule cells and large inhibitory interneurons, Golgi cells. Golgi cells in turn inhibit granule cells. The granule cell axon rises into the outer layer, the molecular layer, where it divides in two to form parallel fibres, which lie parallel to the cerebellar surface and other parallel fibres. Parallel fibres make contact in passing on Purkinje cells, which carry the sole output of the cerebellar cortex.

We attempt to explain neuroanatomy of the granular layer as a strategy that automates the granular layer computation. The computation, in this contention, is the passive result of anatomy (cell morphologies and neuroanatomical architecture) and linear signalling. Speaking generally, neuron-to-neuron transmission involves the complex interaction of a large array of often co-dependent biophysical and other variables which are specific to the cell types making and receiving contact. Yet despite the potential to be dysfunctionally noisy, linear relationships are in fact conserved. There is now a substantial body of evidence that rate-coded information is linearly conserved in communication between cerebellar neurons [1–7], and that cells are adapted for faithful transmission [8–14]. Firing rates that linearly code behavioural and task metrics have been observed ‘at all levels of the cerebellar circuit’ [15, p. 239, citing 30 sources]. There are a number of examples that the cerebellum is adapted to disengage or otherwise compensate for intrinsic non-linear properties of neurons and synapses [12,13,16,17]. This is—we submit—part of a general strategy of isolating the effect of parameters that code information while eliminating or mitigating interference by other variables. Evidence of linear signalling in the granular layer is particularly rich. We include a short review in a dedicated section before §8 to which we cross refer from the main text.

We will argue that the granular layer is in effect the physiological form of a network of linear units which randomly sample firing rates in the afferent cell layer, within topographically defined boundaries. As sampling units have identified anatomical counterparts, anatomy provides model parameters. We field evidence in the main text to explain the derivation of model parameters. Parameter values (typically a range) are usually available from the literature.

Classically, neural network models solve a problem. The nature of the problem—and therefore the function of the network—is a proposal and becomes an assumption of the model. The solution is a computational mechanism; the mechanism is the model. The direction is function → mechanism (the first two steps of Marr’s three levels of analysis; the third step is to consult the evidence for support). We work in reverse. We propose first a mechanism that can explain the evidence, then infer function from the mechanism. In our process, high-level ideas come last. We use modelling in a different role, to quantify and test the ideas by simulating the mechanism. In our approach, the hypothesis and simulation are *both* the mechanism/model. The simulation is the hypothesis in quantified form. While there is a computational element, and the ideas are quantified with a computer simulation, it is not primarily a computational paper.

Note that we do not propose to be mathematically interesting. The heart of the proposal is that the physiological computation is in fact not sophisticated, against expectation—at least, there is a plausible argument that this is the case, which we present as an alternative to current models. For that reason, physiology has a high profile in the paper. The sophistication of the biological design is found in the fact that physiology is adapted to isolate computationally relevant parameters (and computations themselves) from interference by other variables. Biophysical events which accomplish this result are indeed complex. However, there is no need to include them to run the computation. It is standard practice to use a value to represent firing rates, without modelling the complex mechanics of spike propagation. We extend that convention to dendritic signals.

The next section describes our methodology in more detail. We encourage the reader not to skip §2 because it describes how the computation is related to the physical hardware of the system. After that, the paper is divided into four main sections, each building on prior sections, one each on mossy fibres and Golgi cells, and two on granule cells. The main ideas/proposals are summarized in box 1.

Box 1. **A computational effect of neuroanatomy.**

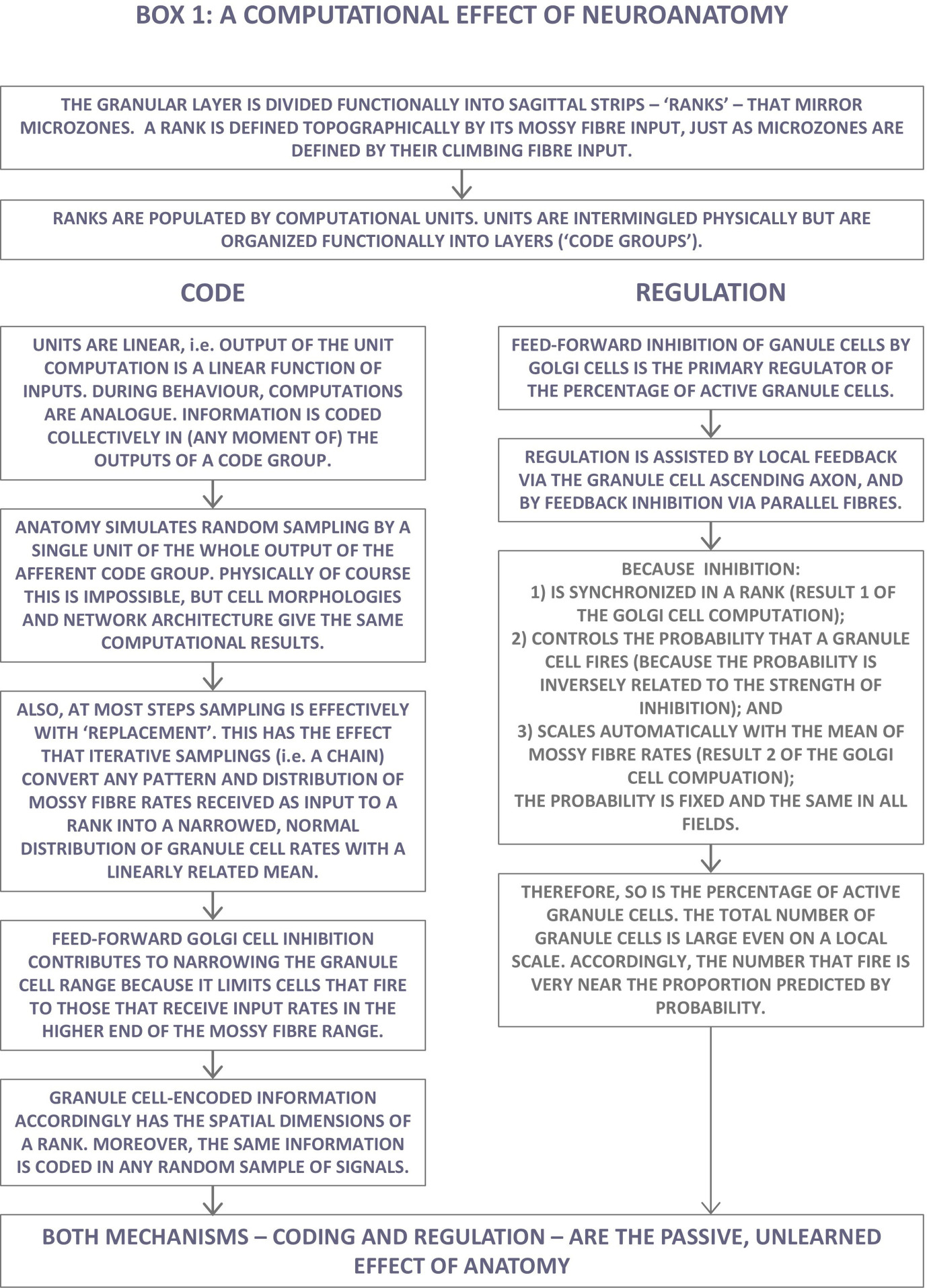

## 2. Methods: the relationship of physiology and model parameters

### 2.1. Summary of methodology

We propose a physiologically detailed hypothesis which we recreate *in silico* in the form of a network of unit functions. Functions are attributed to identified anatomical hardware, connected by known anatomy, and the functions themselves are individually evidenced. In this form, we are able to model large cell networks in high relevant definition, without simplifying the computation (notwithstanding that parameters are not biophysical).

We argue that the computation is a passive effect of anatomy. The simulation provides a demonstration that processing by anatomy provides a mechanism that (i) self-regulates the fraction of granule cells that fire at any time, and (ii) converts input signals into an unexpectedly low-resolution internal code with spatial dimensions.

### 2.2. Computational architecture

The granular layer receives input to the cerebellum and converts it to an internal code. Granular layer anatomy is the physical form of an orderly network of computational units. A unit takes inputs and returns output. Inputs and output represent values (that is, numbers). A unit is the site of biophysical events which relate inputs and output—the unit function.

Physically, the granular layer appears anatomically seamless—a carpet of cells. However, they are wired so that units are organized functionally in layers. The outputs of a layer provide the data sampled (i.e. received as inputs) by units in the next layer.

Computations in a layer run in parallel, continuously, so unit outputs are constantly refreshed, that is, analogue. This occurs in all layers at once.

All layers of unit functions span the network, forming a long, thin strip. All strips are sagittal, and therefore parallel. Information is coded collectively in the concurrent outputs of a layer. The code is a frequency distribution, so that a whole layer, at any given instant in time, codes information that is both indivisible (cannot be subdivided into smaller units of information) and yet coded in any random sample of unit output values. The biophysical form of outputs depends on the layer.

### 2.3. How are units connected?

By anatomy, so we know what the connections are, because anatomy has been reported in detail. The number of inputs to a unit is usually anatomically randomly variable in a reported range. In some cases, we simulate that by generating the number with a distributed probability, and in others we use the mean.

It is unnecessary to know which individual cells contact each other because (biologically and in the model) contact is at random inside topographically defined dimensions. This has the asset for modelling that a target population can be represented by randomly sampling data received as input to a location.

Biologically, a location is a volume with spatial dimensions. *In silico*, we can represent that as a population of unit functions. We know from anatomy (or can derive) the size of unit populations, population ratios, and convergence and divergence ratios.

### 2.4. What determines the shape and size of a population of unit functions?

The cerebellar cortex is anatomically seamless—there are no physical boundaries that divide cells into groups, just functional organization that reflects topography. Microzones are an example [18].

Microzones are divisions of the molecular layer. Mapping studies suggest that the granular layer may have similar organization, but the picture is less clear [19]. However, the morphology and termination pattern of mossy fibres suggest a functional division into strips that may mirror microzones.

**Figure 1.**
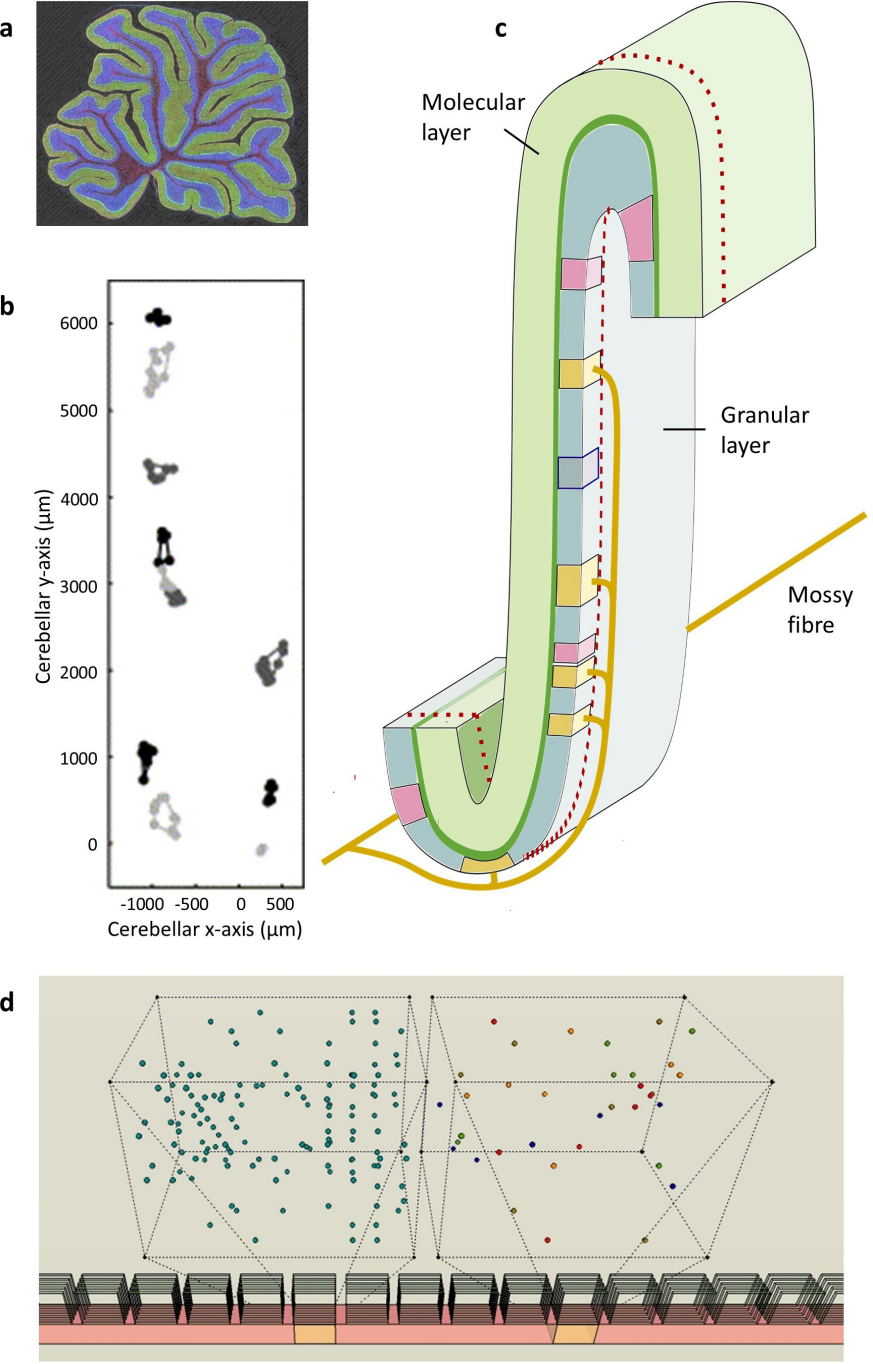
Mossy fibre termination. (*a*) Sagittal section of the rat cerebellum (obtained by multi-photon microscopy, courtesy Tom Deerinck). The outer layer of the cerebellar cortex, the molecular layer, is stained green and the inner layer, the granular layer, is blue. (*b*) Output of a procedure used to group mossy fibre terminals into clusters [20, fig. 6], reproduced with permission. All terminals belong to one mossy fibre, which terminates in two strips. Clusters are shown in different shades of grey. Cerebellar *x*-axis: parallel fibre axis; cerebellar *y*-axis: parasagittal axis. (*c*) Schematic of an area of the folded cerebellar cortex. The strip inside the red dotted line is the width and depth of a microzone but only part of its length. Yellow zones are terminal locations of a single mossy fibre. Pink zones are terminal locations of a second mossy fibre. In reality, zones overlap extensively and terminals are intermingled. The zone enclosed by a blue line has the dimensions of a ‘field’, a nominal division of a rank with the average dimensions of a mossy fibre terminal cluster. (*d*) Section of a microzone viewed obliquely from the cerebellar surface, to illustrate the size and random intermingling of mossy fibre terminals. The wire frame boxes are an exploded view of the orange fields. The right box shows randomly distributed terminals of five mossy fibres (each a different colour). Proportions of terminal size to field size are preserved. The left box shows terminals of 20 mossy fibres (out of approximately 180 that contribute terminals to a field).

Mossy fibres give rise to sagittally extending collaterals, the direction of the long axis of microzones (figure 1). Collaterals branch terminally, and each branch ends in a cluster of terminals, so that a single cell terminates in a spaced-out row of terminal clusters which are always lined up in the same direction (parallel to microzones but in the granular layer) [20–23]. Clusters are separated by a randomly variable distance typically not less than 200 µm [20,21] measured from edge to edge of clusters (so they are separated by a greater distance measured from centre to centre). Terminals are small compared with the average volume occupied by a cluster, and intimately and randomly intermingled with terminals of other mossy fibres.

Accordingly, mossy fibres do not terminate at a point but a region with dimensions: the field occupied by a terminal cluster. A single mossy fibre gives rise to several terminal branches and each of those to several terminals, such that a sagittal strip of the granular layer receives multiple copies of every signal at each of multiple locations.

The dimensions which enclose a cluster (cluster size) are variable (because it depends on the number of terminals in a cluster), averaging 200 µm sagittally × 150 µm mediolaterally [20]. Say we divide the granular layer into strips and strips into ‘fields’ (each perhaps 1/100th part of a strip, but still each containing thousands of granule cells). Fields are average cluster size. Fields are simply nominal, a convenient modelling device. However, strips are functional—the minimum dimensions at which the cerebellum makes any effort to tell signals apart, we submit.

The shape and size of a population of unit functions are a consequence of this topography—the dimensions of a strip where mossy fibre terminals are mixed up at random. This is the first way that mossy fibre morphology is the key to the cerebellar code. All layers of units have these dimensions because they are defined functionally by mossy fibre input, just as microzones are defined by their climbing fibre input.

A sagittal row of 75−100 fields has the area (viewed from the cerebellar surface) of a mid-range microzone [18,24–26]. We term a row of fields a ‘rank’ (strip has been used in the literature to refer to other divisions of the cerebellar cortex).

### 2.5. Derivation of mossy fibre rates used in the simulation

To use the simulation to test if an anatomical computation is feasible, it is necessary to generate input to the system that reflects the effect of mossy fibre terminal branching and terminal clustering, and also that a rank and a field receive input from the correct number of mossy fibres, and that input to a field reflects the fact that cluster size is randomly variable and most clusters straddle field limits. The derivation of mossy fibre rates received as input to a rank and to fields is described in appendix A.

## 3. Mossy fibres

### 3.1. Terminal branching mimics independent sampling by a field of input rates to a rank

We propose that terminal branching of mossy fibre collaterals simulates independent random sampling, by fields, of input rates to a rank. That is, inclusion of a rate in a sample does not affect the probability that it is including in any other sample, also called sampling with ‘replacement’.

If each mossy fibre could terminate in only one field, a mossy fibre ‘sampled’ by one field clearly cannot also be sampled by any others. Termination by a single mossy fibre in a random number of terminal branches, at multiple locations, removes that restriction.

In theory, sampling with replacement would mean any number of fields can receive the same signal, up to all of them. Clearly this is not true—in reality there is an anatomical limit. However, even if there was real replacement, the number of repeat samplings is constrained by a probability distribution—effectively, a cap. If the number of terminal branches per mossy fibre has the same probability distribution, branching has the same result as if firing rates received by each field in a rank were in fact a random sample with replacement of firing rates received by the rank as a whole.

We can test that by calculating the probability distribution in the hypothetical case that mossy fibres rates were in fact sampled with replacement but with a probability (that a given sample contains a given rate) that reflects the physiological number of terminal branches per cell. The probability, *p*, that a given rate is selected *k* times in taking *N* samples, sample size *x,* assuming mossy fibres each give rise to an average of *m* terminal branches, is given by


$$\left[\frac{Nx!}{k!\left(Nx-k\right)!}\right]*{\left[\frac{1}{\left(Nx/m\right)}\right]}^{k}*{\left[1-\left(\frac{1}{\left(Nx/m\right)}\right)\right]}^{Nx-k}$$

where *N* is the number of fields per rank, each receiving innervation by a number of mossy fibres, *x*, given by the derivation of mossy fibre rates in appendix A, and assuming *m* = 5. The results are shown in table 1.

**Table 1.** Probability *p* of *k* repeat selections of a single mossy fibre.

k	0	1	2	3	4	5	6	7	8	9	10	11
p	0.007	0.034	0.084	0.140	0.175	0.175	0.146	0.104	0.065	0.036	0.018	0.008

Although data are scarce, the value of *m* is consistent with what we know about the biological range of the number of terminal clusters per mossy fibre [20,27]. However, some different value would still make the same point. The number is restricted statistically to a small range. The number has the same distributed probability as the number of mossy fibre terminal branches per cell. Physiology in this way mimics sampling with replacement.

### 3.2. Terminal clustering mimics independent sampling by single cells of input rates to a field

Granule cells and Golgi cells both receive contact from a random sample of mossy fibres which innervate a field. We propose that mossy fibre terminal clustering simulates independent random sampling, by individual cells, of input rates to a field. The principle is the same as with terminal branching, but at local scale. Mossy fibre terminal clustering, and random intermingling with terminals of other mossy fibres (figure 1), means that contact on any particular cell does not mean other cells cannot also receive the same signal. Again, to approximate independence, it is only necessary for the number of repeat selections of a signal to have the same probability distribution as if there was actual replacement.

A practical problem with the strategy of terminal clustering is that a target could receive the same signal twice (or more). The risk is mitigated by taking small samples. A sample size of four is small enough for a low risk. Granule cells average four dendrites, each receiving contact from a single mossy fibre terminal, and we estimate that Golgi cells receive an average of four inputs to each of their basal dendrites (see derivation of parameters in later sections). Given *m* × 100 terminals and an independent and equal chance that contact is by any terminal, the probability of an *n*th contact by the same mossy fibre on a single dendrite is:


$$P_{n}=\left[\prod_{x=2}^{n}\left(\frac{m-x+1}{100m-x+1}\right)\right]\times \frac{y!}{x!\left(y-x\right)!}$$

where *y* is the number of inputs to a single dendrite and *m* is the number of terminals per cluster, provided by (for illustration) 100 mossy fibres. Calculated in this way, around one in every 20 samples contains a duplicate, and less than two in a 1000 contain more than one. The actual fractions are smaller because the number of mossy fibres that innervate a field is higher, around 180 (see ‘Derivation of mossy fibre rates’ in appendix A).

### 3.3. The combined effect of terminal branching and terminal clustering

The combined effect is a biological facsimile of simultaneous random sampling with replacement by individual granule cells and Golgi cells of firing rates received as input to a whole rank, notwithstanding that single cells receive fixed contact from a tiny fraction of mossy fibres. This is the second way that mossy fibre morphology is the key to the cerebellar code. Note that it is the population of mossy fibre *rates* that are hypothetically sampled with replacement, and not *signals*. The population of signals includes copies of rates because of branching and clustering.

## 4. The Golgi cell computation

### 4.1. Hypothesis

Despite forming an anatomically seamless carpet of cells, Golgi cells are organized functionally in groups: ensembles. An ensemble is the population of a sagittal row of three fields. This is the minimum functional unit. Golgi cell morphology creates ensemble dimensions. The Golgi cell axon ramifies profusely, giving rise to a dense plexus. The axonal field is sagittally elongated—that is, in the direction of the long axis of a microzone (mean range 650 ± 179 µm by 180 ± 40 µm (in mice [28]))—and is the depth of the granular layer, which it fills vertically. Because of the dimensions and orientation of the Golgi cell plexus, all Golgi cells in a sagittal row of three fields inhibit substantially the whole of the middle field. Conversely, each field defines a functional population of Golgi cells—an ensemble—being those cells with convergent input to that field. A single Golgi cell contacts thousands of granule cells [29,30].

Each mossy fibre terminal is ensheathed by a semi-permeable membrane which restricts neurotransmitter diffusion, a structure termed a glomerulus. Excitation of granule cells and Golgi cells by mossy fibres, and inhibition of granule cells by Golgi cells, all take place here. Fine, beaded Golgi cell axon fibres enter glomeruli, where they inhibit granule cells [29]. ‘In the adult rat cerebellum, each granule cell dendrite receives 2.6 ± 0.55 synaptic contacts from Golgi axon terminals’ [31, p. 791] citing [32].^1^ However, the large majority of inhibition (98%) is by spillover [4], where neurotransmitter released into the synaptic cleft spills out into the glomerular space. Golgi cells release GABA, an inhibitory neurotransmitter. This is detected by high-affinity GABA_A_ receptors located perisynaptically and extrasynaptically on granule cells [33,34]. Even synaptically received signals have most of their effect (i.e. the large majority of charge transfer is mediated) via spillover.^2^

We propose that the anatomical hardware of a Golgi cell ensemble, including glomeruli in the middle field, are the physical form of three unit populations, each a step in the ensemble computation. First, Golgi cell basal dendrites individually each quasi-independently randomly sample mossy fibre rates. Unit output is a sustained dendritic state which is, at any time, a linear function of the sample mean. The second layer of units is the three-field population of Golgi cell somata. Golgi cell somatic charge is a linear function of dendritic states. Golgi cell firing rates linearly reflect somatic charge. Third, middle-field glomeruli each quasi-independently randomly sample firing rates of Golgi cells afferent to the field. GABA spillover is rate proportional, so intraglomerular GABA concentration linearly reflects the mean of input rates.

So, there are three steps of random sampling: the first and third with simulated replacement. There is a statistical effect of this chain of concurrent events. Say a population of values (which can have any frequency distribution) is randomly sampled. There are *n* samples, sample size *m*. If we take the mean (or sum) of each sample, and we take enough samples, the sample means have a normal or near-normal distribution. (This works even if *m* is a range.) The new distribution is centred on the same mean as the sampled distribution, but narrower. This is the result of statistical effects called the central limit theorem. If we then repeat the process, resampling the new data, the result is a further narrowed distribution, preserving the same relationship of the means. We hypothesized that three rounds of sampling are sufficient to synchronize, or narrowly focus, middle-field glomerular GABA concentrations and therefore inhibition of middle-field granule cells.

Ensembles conflate sagittally anatomically. We further hypothesized that the Golgi cell population of a rank functions as a single unit. In this proposal, ensembles in a sagittal strip all return the same output because they all perform the same computation on data randomly sampled from the same mossy fibre distribution. (Unit layers also conflate, so that a functional unit population extends the length of a rank.) The result is that inhibition of granule cells is focused not only in each field but also in a whole rank, with the same mean.

See §7.1 for derivation of parameters not given here. See §7.2 for a discussion of evidence for linear transmission of mossy fibres to Golgi cells.

### 4.2. Results

We first simulated the Golgi cell population of three fields and then the population of a rank (100 fields).

#### 4.2.1. Three fields: the Golgi cell ensemble

**Figure 2.**
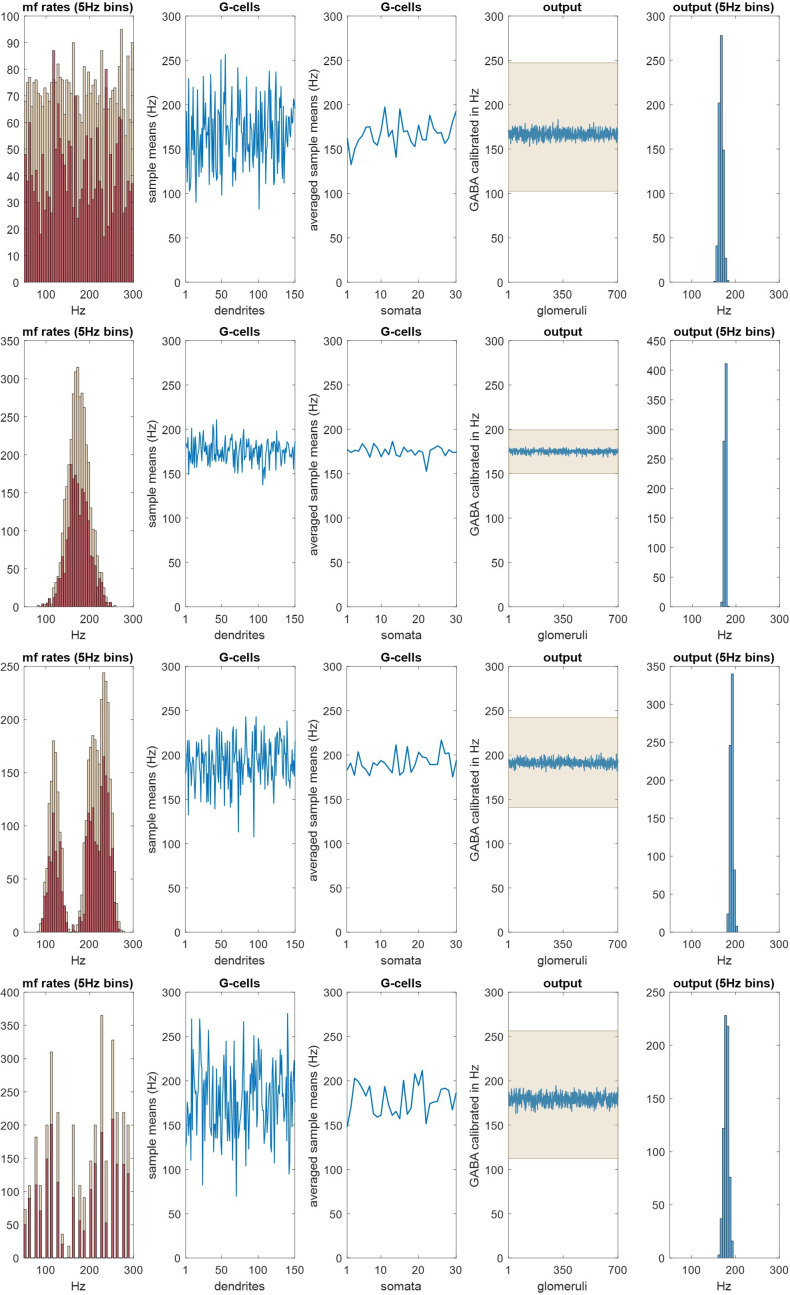
The Golgi cell ensemble conversion. Column 1: we randomly generated (3645) values representing mossy fibre rates with a uniform, normal, twin-peaked or discontinuous distribution, simulating input to a row of 100 fields. Rate data were randomly sampled to derive input rates to each field, and copies added to reflect the fact that each cell afferent to a field contributes a randomly variable number of terminals. See appendix A for derivation of mossy fibre rates. Pale red: input rates to 100 fields without copies added. Dark red: input rates to a row of three fields with copies added. Column 2: the first-column dark red data were randomly sampled 150 times, sample size 4, representing mossy fibre input to a three-field population of 30 Golgi cells, each with five basal dendrites (the mean) which each receive contact from four mossy fibres. See §7.1 for derivation of these numbers. Data are the sample means, representing dendritic depolarization. Column 3: we next randomly sampled column 2 data without replacement 30 times, sample size 5, and took the sample means, representing somatic integration of dendritic signals. Column 4: column 3 data were randomly sampled 700 times, sample size 8−12, reflecting convergent input from three fields of Golgi cells onto 700 glomeruli in the middle field (which receives the output of a three-field Golgi cell ensemble), and took the sample means, representing intraglomerular GABA concentration, the result of rate-proportional spillover. Column 5: the column 4 data presented as a histogram. All rows: column 1 mossy fibre data are converted to narrowly focused inhibition of granule cells. The mean of the column 4/5 data is linearly related to the mean of the mossy fibre distribution. This result is independent of the shape of the original distribution.

Mossy fibre rates sampled by Golgi cells in each field were obtained by sampling from a distribution representing signals received as input to a rank, which were randomly generated subject to constraints on the shape of the distribution, and allowing for mossy fibre terminal branching and clustering, as described in appendix A (figure 2, column 1).

This gave the number of mossy fibre signals, firing rates and the number of copies of each rate received by each field, and therefore input to an ensemble territory, populated by 30 Golgi cells, 10 per field. There are approximately 700 mossy fibre terminals per field. As far as we know (and we propose), contact by mossy fibres on Golgi cells is at random at field scale, i.e. signals received by a single basal dendrite are provided by a random sample of local mossy fibre terminals. The number of physiological samplings is equal to the number of basal dendrites per field; sample size is the number of mossy fibres which make contact on each dendrite. The effect is sustained dendritic depolarization under modulation by a linear function of input rates, which we take as the mean. References for the derivation of Golgi cell ensemble parameters are given in §7.1.

Taking the sample means generates a new set of values (figure 2, column 2) with a smaller range and centred on the mean, which is linearly related to the mean of the sampled distribution. Basal dendritic charge transfer is passive and little filtered [10,35]. Integration of dendritic states at the Golgi soma is represented by taking the mean of dendritic charge, for each simulated cell. Golgi cell firing rates have a linear relationship with the amplitude of depolarizing current [36], suggesting (and we assume) a linear conversion of somatic membrane potential into the Golgi cell firing rate.

The output is a third dataset, Golgi cell firing rates (figure 2, column 3), on the same normalized scale. Each glomerulus in the middle field receives innervation from a random subset of Golgi cells afferent to the field, and accordingly a random sample of Golgi cell firing rates, mimicking replacement. A granule cell extends a single dendrite into a glomerulus, typically receiving synaptic contact from two or three Golgi cells [31]. However, inhibition of granule cells by Golgi cells is almost entirely by GABA spillover into the intraglomerular space [4], via extrasynaptic receptors [33,34], as noted. This effectively increases the Golgi cell to granule convergence ratio, and therefore sample size.

The number of Golgi cells afferent to a glomerulus is unreported, as far as we know. We take it as a random number in the range 8−12, generated for each glomerulus. We tested other ratios to confirm that the ratio does not materially affect the ensemble computation (electronic supplementary material). Spillover concentration is a linear function of the mean (or sum) of afferent Golgi cell rates (additional support is referenced in §7.4) which we take as the mean on a normalized scale.

This generates a final population of values (figure 2, columns 4 and 5), the output of the ensemble conversion. There are 700 outputs, the number of glomeruli in the middle field. All granule cells that extend a dendrite into the same glomerulus receive equal inhibition (of that dendrite).

#### 4.2.2. Ensemble summary/interpretation

There is a progressive narrowing of the data range at each step. Inputs to an ensemble (mossy fibre signals received by a row of three fields) are converted to narrowly grouped and normally distributed outputs (representing GABA concentration in middle-field glomeruli). The narrow output range indicates that inhibition of middle-field granule cells is synchronized, which is to say, statistically constrained (at any moment) to a narrowly focused range. The mean of outputs has a linear relationship with mossy fibre rates, acting through the ensemble conversion. Our interpretation is that granule cells in the middle field receive co-modulated inhibition which tracks the mean of mossy fibre rates. These results are independent of the frequency distribution of mossy fibre rates—input to the system may be in any permutation of firing rates, with the same result.

#### 4.2.3. 100 fields: a rank

**Figure 3.**
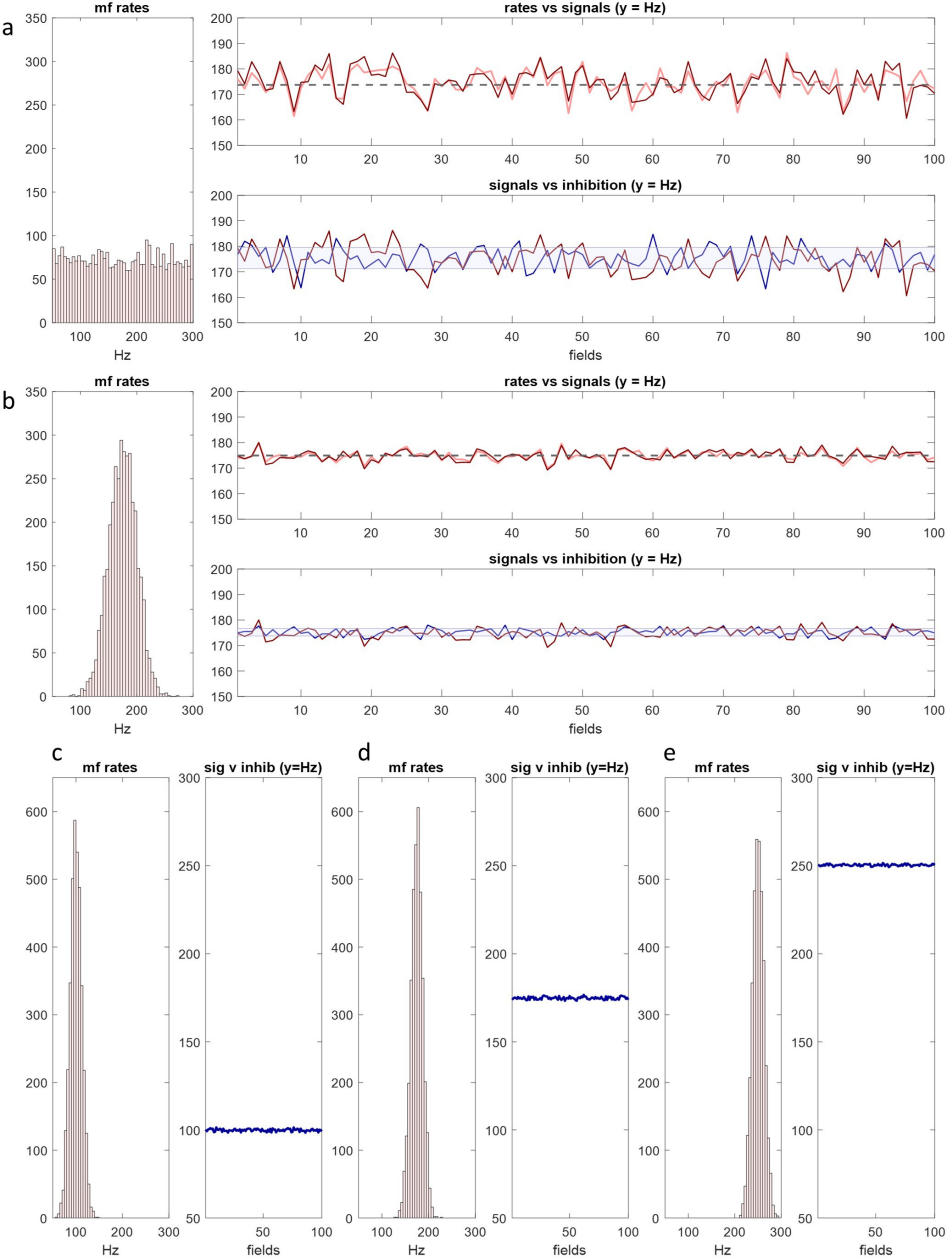
The Golgi cell population of a rank functions as a single unit. (*a*) Simulation of Golgi cells in a sagittal row of 100 fields, taking the mossy fibre distribution on the left as input (see figure 2 and appendix A for derivation of mossy fibre rates). Top right: pink, the mean of mossy fibre input rates to each field; red, the mean with duplicate signals added; dashed grey line, the mean of input rates to the whole rank. Bottom right: red, the same as top right; blue, the mean—for each field—of outputs derived in the same way as figure 2, columns 4 and 5 (the ‘inhibitory field mean’); pale blue, s.d. (*b*) The same as (*a*) but sampling a different mossy fibre distribution. (*a* and *b*) Red data closely track the pink data in top right panels, indicating that duplicate signals (because mossy fibre terminal branches each end in a cluster of terminals) do not skew the mean of firing rates received by a field. Inhibitory field means are tightly grouped: inhibition of granule cells is synchronized in a rank. (*c*) The same simulation. Left: the sampled distribution (mossy fibre input rates to a rank); right: the inhibitory field means (we use the means to compare fields). The *y*-axis of the right panel has the same range as the *x*-axis of the left panel. Panels are tall to improve visual comparison. (*d* and *e*) The same, moving the sampled distribution to the right. Inhibition of granule cells is precisely and linearly coupled to the mean of mossy fibre input rates to the rank as a whole.

We next used the ensemble conversion to derive inhibition of granule cells in a sagittal row of 100 fields, a rank. The results are shown in figure 3. As ensembles all perform the same computation and effectively randomly sample (with anatomically simulated replacement) the same data (the distribution of mossy fibre input rates to the whole rank), they all return the same output, or nearly the same output. There are 700 outputs per field, representing glomeruli, as before. The mean of outputs, calculated for each field, has a narrow range centred on the mean of mossy fibre input rates to the parent rank. Focus varies with the bandwidth of the sampled distribution (mossy fibre rates). A normal mossy fibre distribution with a range of over 100 Hz is converted to a blue range of approximately 3 Hz.

In figure 3 rows (*a*) and (*b*), blue data in the bottom right panel represent the mean, for each field, of outputs. The range of the blue data is centred on the grey dashed line—the mean of mossy fibre input rates received by the rank as a whole— and varies independently of the red data. That is, field-to-field variation of outputs appears to be intrinsic to the mechanism, caused by inherent uncertainty of the outcome of random sampling. However, it is not compounded—random variation of the red data does not flow through to amplified variation of the blue data (in fact the blue data have a slightly smaller range).

#### 4.2.4. Rank summary/interpretation

Golgi cells in a rank act as a single functional unit. Ensembles conflate in the sagittal direction functionally as they do anatomically. As a result, the whole granule cell population *of a rank* receive synchronized inhibition which is linearly related to, and varies with, the mean of mossy fibre rates.

## 5. Granule cell regulation

### 5.1. Hypothesis

Anatomy simulates independent random sampling by a single granule cell of the population of mossy fibre input rates to a rank (§3). There is a competition for influence between mossy fibre excitation and Golgi cell inhibition of granule cells. The competition takes place in the glomerulus. Granule cells receive input to each dendrite from a single mossy fibre. Granule cells have 3−5 dendrites, averaging four (we assume 4, but the result is the same with 3−5). Each dendrite of a single granule cell extends into a different glomerulus. Estimates vary of the number of granule cells that receive contact from a single mossy fibre terminal. Fifty is mid-range [32,37] and consistent with our estimates of the number of terminals and granule cells in a field. Excitation and inhibition compete for influence in a glomerulus. It is unclear if there is always an emphatic winner, but a swing in the dominant direction may be amplified by positive feedback, acting through presynaptic receptors. Activation of presynaptic GABA_B_ receptors on mossy fibres inhibits glutamate release [38], while activation of presynaptic mGluR2 receptors on Golgi cells inhibits GABA release [39]. To fire, a granule cell must receive at least three mossy fibre signals which exceed the inhibitory threshold. In experimental conditions, using stimulation that excites very high mossy fibre rates, granule cells can be induced to fire in response to only one input [40]. However, other accounts suggest one is too few [4,41,42], and three are needed [43,44]. We assume three. Only mossy fibre signals which prevail in the glomerular competition contribute significantly to granule cell depolarization. During behaviour, the competition is ceaseless. The strength of inhibition is synchronized between glomeruli that populate a rank (§4), so that all mossy fibre signals compete, at any moment, against the same inhibitory threshold. The outcome in each glomerulus is quasi-independent of the outcome at other terminals, including other terminals that innervate the same granule cell. To fire, a granule cell must receive a minimum number of mossy fibre inputs that prevail in the glomerular competition. The probability that enough prevail depends, at any moment, on the strength of inhibition. Since inhibition is synchronized, the probability is the same, at any time, for all granule cells. The probability that a given granule cell receives three competitive signals and therefore fires is a function of the percentage of mossy fibre signals that are competitive. Expressed as an equation, the expected number of granule cells that fire in a field is the result of


$$E_{fld}=T\sum_{x=j}^{n}\left[\frac{n!}{\left(n-x\right)!x!}{\left[P\left(e>i\right)\right]}^{x}{\left[1-P\left(e>i\right)\right]}^{n-x}\right]$$

where *T* is the total number of granule cells per field, *n* is the number of dendrites per granule cell, and *j* is the minimum number of dendrites that must receive dominant excitation, *e* > *i*. The proportion of granule cells that fire is near the expected number by the law of large numbers. The percentage of mossy fibre signals that are competitive is always the same because inhibition scales with the mean of mossy fibre rates (because Golgi cells translate mossy fibre firing rates into proportional inhibition). As the percentage is unvarying, so is the probability. This relationship is always conserved regardless of the original distribution (the frequency distribution of mossy fibre rates received as input to the system). So, the probability does not vary either between locations or with time. Since the probability is invariant, therefore so is the proportion of active granule cells.

Note: the idea of an all-or-nothing result of the intraglomerular ‘balance’ of excitation and inhibition has appeared previously [45].

### 5.2. Results

**Figure 4.**
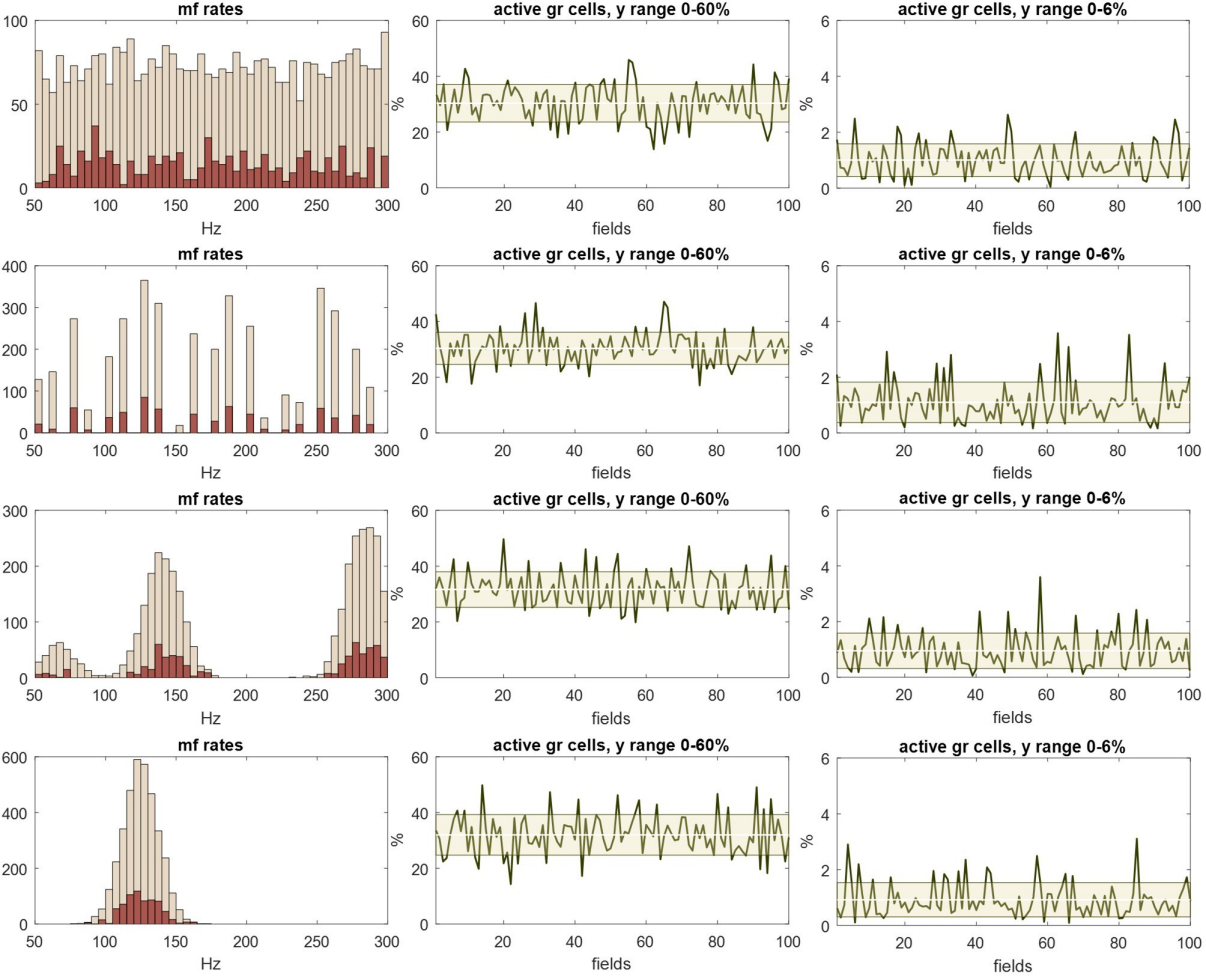
Regulation of granule cell activity by the feed-forward pathway (mossy fibres→Golgi cells→granule cells). We incorporated the Golgi cell ensemble conversion into a simulation of the conversion of mossy fibre signals into granule cell signals (recoding). One of the outputs of recoding is regulation of the percentage of active granule cells. We tested if the recoding computation causes the percentage to be stable, i.e. in the same narrow range in all fields, and whether there is an effect of the frequency distribution of mossy fibre rates. Column 1: mossy fibre firing rates were randomly generated with a uniform, discontinuous, three-peak or normal distribution, to represent input to a row of 100 fields. Rates, and rates received by each field, were derived in the same way as earlier figures. Red data are rates received by a single randomly selected field, including duplicate signals. *y*-axes are scaled to the data—all distributions contain the same number of signals. Column 2: the percentage of granule cells that fire in each field. The Golgi cell computation includes no adjustment to sparsen granule cell activity. The white line and shaded area are mean and s.d. Regulation is poor: there is wide variation from field to field. Column 3: the same as column 2 with an adjustment (a co-efficient) to sparsen granule cell activity. *y*-axes have 1/10 the range of column 2. Regulation is strict: the percentage of active cells is confined to a narrow range. This result is independent of the sampled (i.e. mossy fibre) distribution, suggesting that regulation is independent of fluctuation of mossy fibre rates with time. Nonetheless, field-to-field variation relative to the mean is significant. Homeostatic local fine tuning may be provided physiologically by feedback via granule cell ascending axon contact on Golgi cells, not represented in the simulation.

The model compares mossy fibre signal frequency with inhibition at each of four dendrites of each of 8750 granule cells per field, in each of 100 fields. Mossy fibre input rates to each field are derived, and duplicate signals added, in the way described earlier. Inhibition—outputs of the ensemble conversion, 700 per field—is provided by the Golgi cell model (which takes the same mossy fibre distribution as input). Mossy fibre signals are randomly sampled. Output of the model is the number of granule cells that fire in each field and the mean of mossy fibre rates they each receive (the sample means). The number that fire in each field (in a simulation representing a moment of activity) is shown in figure 4.

Feed-forward inhibition (mossy fibre → Golgi cell → granule cell) reliably constrains the proportion of active granule cells to a narrow range if the fraction that are active is small. The range of variation of the number between fields and between simulations is inversely proportional to the fraction that are active, suggesting that, if primary regulation is provided by feed-forward inhibition (and we are correct about the mechanism), the physiological number is low— termed sparse code. We would nonetheless expect to see greater precision, suggesting the physiological presence of a supplementary mechanism(s) that is not represented in the model.

A local feedback mechanism may be provided by granule cell ascending axon contact on Golgi cells. The ascending portion of the granule cell axon makes synaptic contact on local Golgi cells, on basal dendrites [10]. A Golgi cell receives approximately 400 functional synaptic contacts from this source, around 25% of the granule cells that a Golgi cell receives contact from in total. During sustained mossy fibre discharge, synapses from local granule cells contribute equivalent charge to Golgi cells as mossy fibre synapses and ‘display similarly fast kinetics’ [46]. Short-latency inhibitory feedback may add homeostatic fine adjustment to feed-forward regulation at local level.

It is a long-standing idea [47] that granule cell activity may be regulated by feedback via parallel fibres, which contact Golgi cell apical dendrites. We comment on regulation by feedback inhibition in appendix B.

Potent local regulation is necessary for active granule cells to be randomly and therefore evenly distributed among the general population. Uniform density of active granule cells is important for mossy fibre information to be faithfully coded by granule cells. The granule cell code is the subject of the next section.

The results are independent of:

- the frequency distribution of mossy fibre rates;
- whether mossy fibres are all active or some are active and some are not; and
- the permutation they are active in (which ones are active and which are not).

## 6. The granule cell code

### 6.1. Hypothesis

Granule cells code information in their collective activity, in functionally defined groups (‘code groups’). Code groups are functionally defined by the topography of mossy fibre input to the cerebellum, as Golgi cell networks are. The output of a rank is the smallest unit of granule cell encoded information. Granule cell encoded information, accordingly, has spatial dimensions, the dimensions of a code group. Information is coded in the frequency distribution of granule cell firing rates. Therefore, the same information is coded, at any moment, in firing of any random sample of co-active cells, provided the sample is large enough. Because granule cells are very numerous, even sparse code is sufficient for a Purkinje cell to receive enough. The pattern of active and inactive granule cells/parallel fibres, and how firing rates are distributed among active cells, code nothing.

A granule cell receives contact from a single mossy fibre to each dendrite. Sample size is therefore four (on average), and the number of samples per field is the number of granule cells, which we estimate at 8750 (see appendix A). The unit function is only operative if a cell receives at least the minimum number of competitive mossy fibre signals. Somatic depolarization is a linear function of the mean (or sum) of competitive input rates (evidence is discussed and referenced in §7.3). Dominant signals displace a significant effect of inhibition. Derived in this way, mossy fibre rates reliably predict granule cell rates at collective level, by the central limit theorem. The range of granule cell rates is narrower, centred on the mean and attracted to a normal distribution. The mean of the new distribution is a linear function of the mossy fibre mean. This result is repeated in all fields. As all fields randomly sample, at any time, the same mossy fibre distribution, and perform the same computation, they all return the same granule cell code.

### 6.2. Results

**Figure 5.**
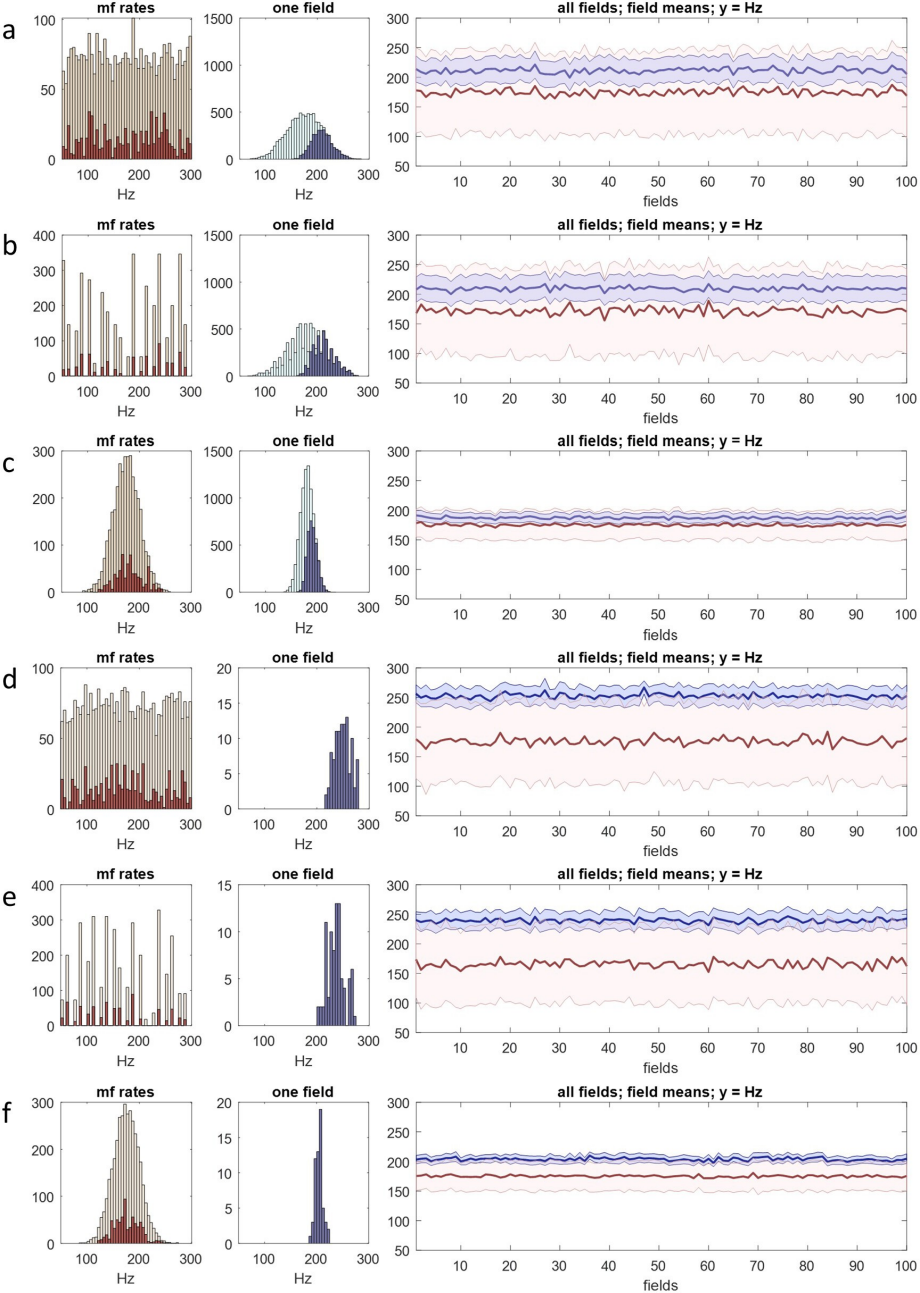
A rank functions as a single unit. The same simulation as figure 4 but data are granule cell firing rates or derived from granule cell firing rates. Column 1: mossy fibre rates were generated in the same way as earlier figures (the number of signals is the same in all rows). Column 2: pale green: sample means—the mean of input rates to each granule cell in a single field selected at random; blue: sample means of the subset of granule cells which meet the conditions to fire. In rows (*a*–*c*), approximately 30% fire. In rows (*d*–*f*), approximately 1% fire, *y*-axes are rescaled and green data are omitted. Column 3: red: mean of mossy fibre input rates to each field in a rank, including duplicate signals; pink: s.d. of mossy fibre input rates to each field; blue: field means, i.e. the mean, calculated for each field, of the average of input rates received individually by granule cells which fire (the mean of the sample means); pale blue: s.d. of the sample means. In all tested conditions, the field means always converge towards a straight line which is a fixed distance from the mean of the sampled distribution (mossy fibre rates in the first column). The amount and direction of field-to-field random fluctuation of the blue data is independent of local fluctuation of the red data, and in a smaller range, indicating that untidiness (random variability) of the data viewed narrowly is not compounded at collective level.

**Figure 6.**
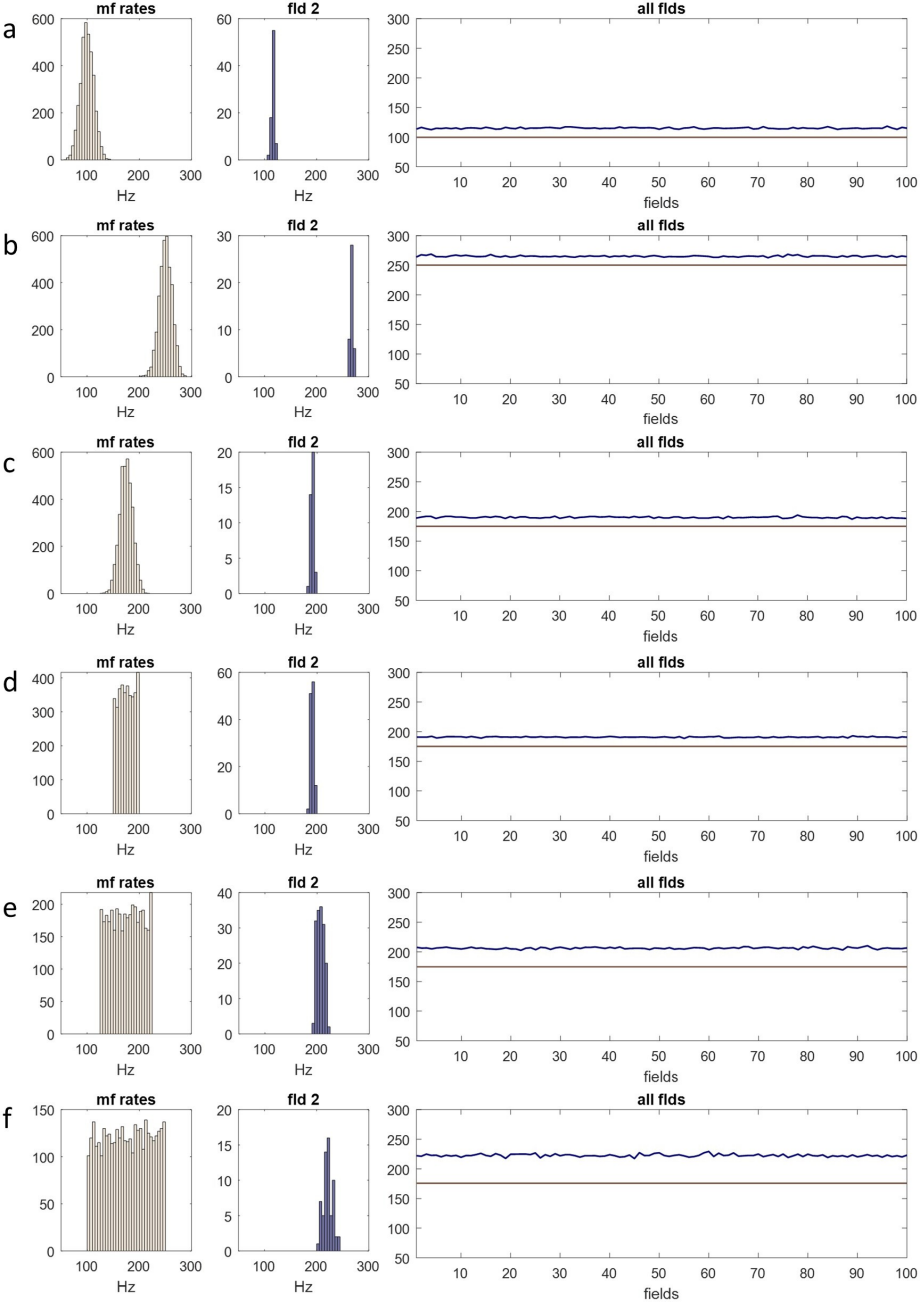
Granule cells collectively code mossy fibre rate information. Column 1: mossy fibre rates were generated in the same way as earlier figures, with either a normal (s.d. 12.5) or uniform distribution, representing input to a row of 100 fields, with copies added—the sampled distribution. Column 2: histogram of granule cell firing rates in a randomly selected field, obtained in the same way as in the bottom three rows of figure 5. Column 3: grey line: the mean of the sampled distribution; blue line: field means. (*a*–*c*) The field means are precisely and linearly related to the mean of mossy fibre rates. Blue data approach a straight line which is parallel to the grey line at a mean distance of 15 Hz (s.d. 1.21 Hz). (*d*–*f*) If the shape and mean of a uniform sampled distribution are unchanging, the distance between the blue and grey lines is linearly related to the range of the sampled distribution.

We ran the same simulation as §5 to test whether, and how, mossy fibre rates are related to granule cell rates, and if fields in a rank all have the same output. As before, the model compares mossy fibre rates with inhibition at each of four dendrites of each of 8750 granule cells per field, in each of 100 fields. Mossy fibre signals received as input to each field are derived in the same way, described in appendix A, and inhibition is provided by the Golgi cell model. We know (or can derive) from reported anatomy the size of unit populations, population ratios, and convergence and divergence ratios. The results are shown in figures 5 and 6. The outputs of the model are the number of granule cells that fire in each field and the mean of input rates to each active cell—the sample means. The mean of the sample means for each field is the ‘field mean’.

The narrowing and predicted shape of the granule cell frequency distribution is evident in all conditions. The field means approach a straight line, the test that a rank operates as a single functional unit. When mossy fibre rates are normally distributed (s.d. 12.5 Hz), the granule cell distribution is very narrow (and normal if rates are counted in narrower bins) and the field means are very close to a straight line. The field means follow the mean of mossy fibre rates at a fixed distance. In all tested conditions, granule cell rates have a condensed range. The shape of the granule cell distribution is attracted to normal, regardless of the shape of the mossy fibre distribution. In a rank, it is very near normal. At single field level, nearness to a normal shape is affected by the shape and range of the mossy fibre distribution. There is a statistical relationship of mossy fibre and granule cell rates even though a single granule cell receives a tiny fraction of mossy fibre rates, and each field receives only a small fraction of mossy fibre signals received by a rank.

What may we infer from that? An anatomically detailed network of linear units in the form described reliably converts mossy fibre rates to a well-predicted granule cell distribution. The simulation assumes, because we propose:

- computational units are linear;
- contact between cells in the granular layer is at random below a scale threshold set by range and morphology;
- sampling mimics replacement, an inference from anatomy; and
- the frequency distribution of granule cell firing rates is code.

The function of the simulation is to test/demonstrate that given these assumptions there is feasibly a computational effect of granular layer cell morphologies and network geometry. If the proposals are correct, the output of a rank is the minimum resolution of granule cell information. Information accordingly has spatial dimensions defined by the topography of mossy fibre input to the granular layer of the cerebellar cortex (just as microzones are defined by the topography of climbing fibre input to the molecular layer). Those dimensions are the dimensions of a granule cell code group. Below that scale, detail is discarded—there is no finer level of discrimination. Activity in any smaller part of that region is simply a physically smaller representation of the same information. The minimum resolution of information is not the smallest unit of code.

The same information is coded in any random sample of granule cell signals, over a minimum number. It is immaterial (i.e. codes nothing) which particular cells are active or in what pattern, or which cells fire at what rates, or even what particular rates those are in any individual case.

## 7. Support for linear transmission

There is a growing body of evidence of linear communication in the granular layer, indicating that unit functions are linear. This section, except the first subsection, is a short review.

### 7.1. Derivation of some Golgi cell ensemble parameters

Four Purkinje cells fit in a field, based on field size, thickness of the Purkinje cell dendritic plate—9 µm [48]—and the distance between them—approximately 40 µm [49]. ‘In man, the ratio [of Purkinje cells to Golgi cells] is 1 ∶ 1.5, while in the monkey and cat it is almost 1 ∶ 1.9 and in the rat 1 ∶ 3.3’ [50]. Possibly, estimates are low because smaller Golgi cells at deeper level were not counted [49, p. 121]. We take it that there are 10 Golgi cells per field, a ratio of 1 : 2.5 with four Purkinje cells.

Contact by mossy fibres on a Golgi cell is on basal dendrites. Four to six basal dendrites arise from the Golgi cell soma. We assume five, so a population of ensemble-grouped Golgi cells has a combined total of 30 × 5 = 150 basal dendrites. The number of mossy fibres which contact a Golgi cell is unreported. Dendrites radiate from the cell body. A single dendrite has a range of 50−100 µm [49]. The longest dimension of a field—which contains approximately 700 mossy fibre terminals—is 200 µm. We estimate the mean number of mossy fibres that contact a single dendrite is four, so 20 per cell, or approximately 3% of the population of terminals (or 0.6% per dendrite).

Glomeruli individually receive innervation from a random sample of Golgi cells afferent to a field. It is unknown how many Golgi cells innervate a glomerulus.

### 7.2. Linear transmission of mossy fibres to Golgi cells

#### 7.2.1. Main discussion

Golgi cells are large interneurons whose cell bodies and basal dendrites lie in the granular layer. Mossy fibres directly contact Golgi cell basal dendrites. Golgi cell firing frequency increases linearly with the amplitude of depolarizing current [36, p. 845]. ‘Golgi cells can follow peripheral signals in a continuous fashion, modulating their frequency with the intensity of the stimulus [citing [51,52]]’ [53, p. 3649]. As a result, during movement, the ‘output of Golgi cells varies smoothly as a function of input.…They are capable of modulating their output to reflect their input with little noise or signal distortion’ [11]. ‘Sensory-evoked Golgi-cell inhibition scales proportionally with the level of mossy fibre excitatory synaptic input’, such that inhibition reliably conveys mossy fibre rate information [3]. On this evidence: mossy fibre rate information is conserved proportionally in Golgi cell firing rates.

Golgi cells extend their basal dendrites into glomeruli [29,54]. Contact on them by mossy fibres is multi-synaptic [49], contributing to a reliable, rapid (submillisecond) response. The fast rise time and weak distance dependence of the amplitude and timing of Golgi cell excitatory postsynaptic currents evoked by mossy fibre signals, suggests that basal dendrites introduce relatively little filtering [10,35], as expected for large-diameter dendrites. We note that the pause in Golgi cell firing following a discrete stimulus under anaesthesia [3,55,56] disappears from Golgi cell recordings during locomotion [52].

During behaviour in freely moving animals, mossy fibre activity is dense: a high fraction of mossy fibres are active [57]. Both mossy fibres [58–60] and Golgi cells [52] have been reported to fire with a sustained, time-varying signature. In the behaving animal, Golgi cell basal dendritic membrane potential is a continuous variable under modulation by sustained inputs, we submit. As charge transfer to the soma is passive, polarization of the soma is likewise sustained, under modulation by dendritic states.

It is worth noting that very high mossy fibre rates can be generated in experimental conditions. However, the typical physiological range is 50−300 Hz [59]. In the simulations, we use the physiological rates, within stated constraints on the shape of the distribution.

#### 7.2.2. Is there other control of Golgi cells?

Do Golgi cells receive a significant influence of input from other sources? The evidence is incomplete but currently suggests other influence is absent or weak. Contrary to early reports, neither Purkinje cells nor climbing fibres contact Golgi cells (see [61] who give references), and only a modest minority of inhibitory inputs to Golgi cells (if any) are from molecular layer interneurons [62], which generate weak synaptic currents [63], consistent with either extremely weak or wholly absent innervation [64]. There is conflicting evidence whether Golgi cells make inhibitory synaptic contact on each other [64,65]. Our simulation does not include an effect on Golgi cell firing by other sources of input.

#### 7.2.3. Golgi cell oscillations

Golgi cells fire autonomously. Under anaesthesia, firing falls into a slow oscillating pattern [66]. Under excitatory synaptic input, however, this disappears [65]. Anaesthesia ‘has a strong influence on spontaneous activity of Golgi cells’ [61]. Discharge at low rates with irregular timing under anaesthesia [67] is replaced by higher rates with more regular timing without anaesthesia [51,52,68]. This paragraph is a cautionary note about evidence obtained under anaesthesia, which has been used to argue that oscillations provide a form of signalling.

### 7.3. Linear transmission of mossy fibres to granule cells

There is significant evidence that granule cell firing rates are linearly related to input rates, but also evidence that has been given a conflicting interpretation. We take them in turn.

The short and equal length of granule cell dendrites would suggest light and equal filtering. The mossy fibre-granule cell connection has a range of adaptations which support high-fidelity transmission of high frequency signals across a wide bandwidth [8]. Faithful transmission of rate information is reported [1,40]. Vesicle release and replenishment are fast [9,12] and postsynaptic AMPA receptors operate in their linear range [12], where they are resistant to desensitization [69]. Multiple contacts are made by a mossy fibre with each granule cell [32]. Conversion of depolarizing somatic charge into granule cell firing is a simple function, such that granule cells ‘have a relatively linear and uncomplicated conversion of depolarization level to spike rate’ [42, p. 2393 ].

Glutamate spillover enhances precision and reliability of transmission [12,70]. During behaviour, at sustained physiological mossy fibre rates, there may be a build-up of intraglomerular glutamate, increasing the relative influence of spillover in a balance with synaptic transmission, assisted by short-term synaptic depression. In these conditions, spillover may dominate, as it dominates inhibitory transmission [4,71]. Spillover from multiple release sites (200–400 at a single mossy fibre terminal) is plausibly sufficient to mitigate variability of vesicular size, release probability and the number of release points at synapses made on any individual cell, increasing fidelity and equality of excitation of granule cells.

Against that, there are reported to be heterogeneous mossy fibre-granule cell synaptic weights. Different strength and short-term dynamics of the mossy fibre to granule cell connection have been reported in vestibular slices, with GABA_A_ receptors blocked. The amplitude of the response, measured at the soma, is reported to be statistically correlated to the source of the mossy fibre that is stimulated [72], suggesting that the strength of the connection depends on the source.

However, the presence of weights does not necessarily mean that the function is to modulate firing rates. Rather, the authors themselves suggest that the function may temporally code the source of inputs because different combinations have different response onset times. An alternative, which they also mention, is that some inputs may be in a supporting role, reminiscent of ‘driver’ and ‘modulatory’ signals in the thalamus and cortex [73]. Feasibly, signals in a supporting role must be present for the postsynaptic granule cell to fire, but do not affect the granule cell firing rate.

Heterogenous granule cell subtypes have been inferred from recordings of granule cells in rat slices, from the finding that when stimulation is extended beyond 500 ms, firing of most cells changes, either slowing down or (in fewer cases) speeding up. However, the significance is not clear. Granule cells typically fire in short bursts—shorter than the duration of the stimulation tested, so perhaps not long enough for the effects observed. Short granule cell burst duration may be because a granule cell normally does not meet the conditions to fire for long, because few fire at any time, and the permutation of active and inactive inputs to a single granule cell, and the firing rates of active inputs, are both in a state of restless change. Also, excitation of granule cells receives a concurrent regulatory influence of a network of Golgi cells which populate a region which receives 100 s of time-varying mossy fibre signals at any time (so, not the conditions tested). Confirmation of functional significance of adaptive firing in experimental conditions is outstanding.

### 7.4. Linear transmission of Golgi cells to granule cells

The hypothesis includes the proposal that inhibition of granule cells is, at any time, linearly related to the mean firing rate of Golgi cells afferent to each glomerulus. In support, we cite evidence that GABA spillover into the intraglomerular space is proportional to afferent Golgi cell rates and that inhibition of granule cells is almost exclusively mediated by spillover, and therefore linearly reflects the intraglomerular concentration of GABA. Citations appear in the Golgi cell section but for convenience they are repeated here.

Fine, beaded axon fibres enter glomeruli, where they inhibit granule cells [29]. ‘In the adult rat cerebellum, each granule cell dendrite receives 2.6 ± 0.55 synaptic contacts from Golgi axon terminals’ [31] citing [32].^3^ However, the large majority of inhibition (98%) is by spillover [4], where neurotransmitter released into the synaptic cleft spills out into the glomerular space. Golgi cells release GABA, an inhibitory neurotransmitter. This is detected by high-affinity GABA_A_ receptors located perisynaptically and extrasynaptically on granule cells [33,34]. Even synaptically received signals have most of their effect (i.e. the large majority of charge transfer is mediated) via spillover.^4^

Golgi cells fire [67] at a time-varying rate in the behaving animal, so that a glomerulus receives continuous input. As a result, there is a sustained build-up of glomerular GABA during behaviour [74] at an adjustable concentration controlled by Golgi cell firing rates [71]. Signalling by spillover is sometimes assumed to be ambient and slow. However, the action of glomerular GABA spillover has a fast phasic component—not as fast as synaptic transmission (approx. 1 ms) but with a rise time of only a few milliseconds [71]. Unlike the spiky appearance of synaptically induced inhibitory postsynaptic currents, spillover [3] generates a sustained outward current.

## 8. Discussion

### 8.1. Summary

Network models are often a simplified representation of physiology to provide a substrate for sophisticated computations. We propose the reverse: physiology is highly adapted to provide a substrate for straightforward computations. In summary we propose:

1. there is a computational effect, unaided and unlearned, of the combination of cell morphologies, network architecture and linear units, for which the granular layer is highly adapted;
2. physiology is the physical form of a network of linear units organized in layers. Units randomly sample outputs of the afferent layer. Code groups communicate by a facsimile of independent random sampling. Anatomy mimics random sampling with replacement by a single cell of the entire population of firing rates in the afferent cell layer; and
3. this has two functions. It regulates the fraction of granule cells which fire concurrently and converts mossy fibre firing rates into an internal code.

Regulation
4. Golgi cells, working together, convert mossy fibre firing rates ad hoc into inhibition of granule cells which tracks the mossy fibre mean and which is synchronized in a rank (i.e. statistically constrained in all fields to the same narrowly focused range);
5. there is a competition in each glomerulus between mossy fibre excitation and Golgi cell inhibition of granule cells. To fire, a granule cell must receive a minimum number of dominant excitatory signals; and
6. the probability that a cell fires depends on the local relationship of inhibition and the mean of mossy fibre rates. Since all granule cells in a rank (1) randomly sample the same distribution of mossy fibre rates; (2) receive synchronized inhibition; and (3) inhibition tracks the mossy fibre mean, they all have the same probability that they fire, and the probability is fixed.

Code
7. Data processing exploits statistical effects of random sampling such that the frequency distribution of granule cell rates is reliably (because statistically) well-predicted by mossy fibre rates;
8. high-dimensional mossy fibre information recodes as a very low-resolution granule cell code. Granule cell code groups function as a single unit, coding information in their collective activity;
9. accordingly, granule cell-coded information has spatial dimensions, the dimensions of a code group. As code groups are long and thin, information has a striped pattern viewed from the cerebellar surface;
10. code groups span the cerebellar network in the sagittal direction, from side to side, mirroring microzonal organization of the molecular layer. Like microzones, they are defined functionally, by their input from outside the cerebellum;
11. the same information is coded in any random sample of code-grouped granule cell firing rates. In that sense, the code is homogenized below group dimensions; and
12. the shape and dimensions of the granule cell code are functional (discussed below).

### 8.2. Commentary

#### 8.2.1. General commentary

Speaking generally, it is difficult to reconcile evidence relating to synaptic learning with direct and indirect evidence of linear synaptic transmission. Historically, there has been strong emphasis on learning [75,76] and an implicit inference from mostly *in vitro* work that synaptic changes are functional. In the learning dominated view, the aim of further work is to understand how plasticity at multiple sites is related, and related to function.

We present an alternative where the focus is to explain cell morphologies and network architecture in the light of evidence of linear communication between cerebellar cells, synaptic adaptations reported to conserve rate coded information, and linear relationships between cerebellar cell firing and behavioural metrics. We propose there is a computational effect of anatomy, given linear signalling.

This has merits. It has good explanatory value—cell morphologies, granular layer network geometry, cell ratios, spatial organization, the shape and size of dendritic and axonal territories, convergence and divergence ratios, random contact and the probability that contact is made, the duplication of mossy fibre signals as a result of terminal branching and because branches each end in a cluster of terminals, the structure and physiology of the glomerulus, and linear signalling (including evidence that synaptic connections are adapted for high-fidelity transmission)—are all accounted for.

We propose—as artificial neural network models do—that signals traffic flows through computational units organized in layers, but with the difference that our units are matched with physiological hardware, and units form functional populations defined by well-known physiology.

As we note in §1, we work in reverse. We infer a mechanism from the evidence and then infer function from the mechanism. High-level, functional justification for the mechanism is more a closing argument than an opening position. We discuss the functional significance and rationale in the remainder of §8. We make three main arguments. In all three, the form of the granule cell code solves a problem. The first problem is how an anatomically seamless tangled mat of cells is organized functionally with a view to convert high-dimensional external signals into coherent firing of microzone-grouped Purkinje cells. The second is that—against expectation—the pattern in which parallel fibres are active may not in fact be reliably or perhaps ever replicated. Theory has previously addressed this problem by making assumptions that may not be safe. The first paragraph of the paper comments on this. The third problem is how insentient cerebellar circuits are able to process input data usefully with what seems to be little or no information about where it is from or what it represents.

#### 8.2.2. A homogenized code may coordinate Purkinje cells

The sacrifice of data resolution on entry to the cerebellum is functional, we submit. Microzones lie at right angles to parallel fibres and parallel to code groups. The statistical form of the granule cell code means the same information is received by all locations of sagittal plane, so that microzone-grouped Purkinje cells can receive the same information without receiving any of the same signals. An estimated 350,000 parallel fibres pass through a Purkinje cell dendritic territory [77–80]. If even only a small fraction of parallel fibres is active (say, 0.5%), and allowing also for the fact that only one in two make contact and that a majority of synapses are severely depressed or silent [81,82], a single Purkinje cell still receives around 200 parallel fibre signals at working synapses at any time. A granule cell code in this form provides a means to modulate firing of microzone-grouped Purkinje cells in unison, ultimately under control of rate-coded input to the system. There is evidence that Purkinje cells linearly code the strength of granule cell inputs [5–7] and linearly code behavioural metrics [2,83–86].

#### 8.2.3. Patterns may not be repeated

Recoding in the granular layer may be a physiological strategy to mitigate variable biological performance, a practical problem for replicable biological information capture. Modelling often assumes that code is replicable—that is, a repeat of the same data has the form of a repeat of the same neuronal activity. The traditional cerebellar learning model [47,87,88] relies on (and assumes) replicability for signals to match up with the correct training-modified parallel fibre synaptic weights.

In fact, it is not clear that input to the cerebellum is ever replicated. Around 4000 mossy fibres innervate the region that supplies parallel fibre input to a single Purkinje cell. It is not known if conditions upstream consistently (or perhaps ever) generate a repeat of the same inputs. A related issue is that neurons and synapses are not standard components, even of the same type. Indeed, the same cell may sometimes behave differently on different occasions in the same conditions. Third, the granular layer is designed to quasi-randomize the pattern in which granule cells/parallel fibres are active. To achieve that result, any differences, in any combination, of the pattern of active inputs, the rates they fire at, and the distribution of rates among active cells, are converted to an amplified effect on the pattern of internal activity. In the alternative model, replication of the pattern of active granule cells is unnecessary to code mossy fibre information because information is coded in rate statistics and not the pattern of active cells.

#### 8.2.4. Working ‘blind’

An issue for theory generally is that biological networks do not seem to have much information about the signals they receive. Input to the cerebellum is eclectic and in multiple modalities. In some cases, a single granule cell can receive multi-modal input. In the traditional cerebellar model, supervision solves this problem. It is unnecessary for the cerebellum itself to discriminate because it is an assumption of the model that externally sourced instruction signals train the correct response.

We propose instead that the solution is to sacrifice the resolution of high-dimensional input data. Source and modality data are disregarded below a topographically defined scale threshold. It is therefore unnecessary to discriminate below that scale. Mapping studies show that mossy fibre signals (evoked by cutaneous stimulation) share the topographical organization of climbing fibres [19,89,90]. At sub-topographical dimensions, we submit, signal source and type are simply discounted. Therefore, networks do not need to be adapted to the source or type of input they receive—anatomically, networks are interchangeable, so network design can be modular. This feature allowed basic cerebellar wiring to be highly conserved through dramatic upheavals of the vertebrate phenotype [91,92].

## Glossary

**Defined terms:**

**Field:** a nominal division of the granular layer measuring 200 µm (sagittal) × 150 µm (mediolateral), the average dimensions of the area enclosing a mossy fibre terminal cluster.

**Field mean:** for each field, the average of the mean of mossy fibre input rates to each granule cell which meets the conditions to fire.

**Rank:** a sagittal row of 100 fields.

General nomenclature

**Glomerulus:** each terminal is ensheathed in a semi-permeable membrane which restricts neurotransmitter diffusion. Excitation of granule cells and Golgi cells by mossy fibres, and inhibition of granule cells by Golgi cells, take place in a glomerulus.

**mossy fibre terminal branch:** mossy fibre axon collaterals give rise to several terminal branches whose endings are separated by a minimum distance and strictly sagittally aligned.

**terminal:** a mossy fibre rosette.

**terminal cluster:** each mossy fibre terminal branch ends in a cluster of terminals. The number of terminals per cluster is randomly variable, with an average of 7−8.

## Data Availability

The code can be accessed here [93]. It is associated with this title, an earlier version of the submitted article: What if...? Computational anatomy in the granular layer of the cerebellar cortex. It can be downloaded in Firefox and Safari. There are sometimes issues with Google. Supplementary material is available online [97].

## Footnotes

^1^
Data are sparse. The original data are an estimate derived from visual inspection of slices 30 years ago so far without later corroboration, except from [31], who derive a lower estimate but propose the disparity is because their estimate is too low.

^2^
Because of the long duration of inhibitory postsynaptic currents (IPSC)s generated by spillover (recorded in slices), the total charge carried is three times that of IPSCs generated by directly connected terminals [31].

^3^
Data are sparse. The original data are an estimate derived from visual inspection of slices 30 years ago so far without later corroboration, except from [31], who derive a lower estimate but propose the disparity is because their estimate is too low.

^4^
Because of the long duration of IPSCs generated by spillover (recorded in slices), the total charge carried is three times that of IPSCs generated by directly connected terminals [31].

## Appendix A. Derivation of mossy fibre rates

### A.1. Rationale

We run the computation *in silico*. We represent the granular layer as a network of unit computations organized in layers, as described. Support for the size of unit populations, population ratios and convergence and divergence ratios is cited in context, in the relevant section of the main text. Support for linear unit functions is discussed in the section before §8.

The simulation is not an additional step in ideas or detail. It is a quantified form of the hypothesis. The hypothesis describes a computational mechanism; the simulation performs the computations. The purpose of the simulation is to confirm that the mechanism is feasible, with plausible evidence. This section describes the derivation of mossy fibre rates received by a rank and by fields taking account of terminal branching and terminal clustering, the randomly variable number of terminals per cluster and random variation of the number of terminals in a cluster which fall inside and outside a field when a cluster straddles a field boundary.

The frequency distribution of mossy fibre rates received as input to a granular layer rank is unreported. We used a large bandwidth in the physiological range and varied the shape of the distribution to confirm that the operation of the mechanism does not depend on the input to it. The shape of the mossy fibre distribution is included in all figures.

### A.2. The number of mossy fibres that innervate a rank

Each field receives a random sample mossy fibre firing rates received as input to a rank. To simulate that, we need the size of the sampled population—the number of mossy fibre signals received by a rank—and sample size, the number received by a field.

Despite the repeating ‘crystalline’ architecture of the cerebellar cortex, at local level there are anatomical random variables. Variables are: (i) the number of mossy fibres that innervate a field, (ii) the number of terminals they each end in, and (iii) the number of those that are inside field limits.

The number of mossy fibres afferent to a field has been estimated at 100 [20]. However, that number is a field-sized fraction of the general population. The actual number is larger because mossy fibres end in a dispersed group (cluster) of terminals, and most groups straddle field limits.

We work from an estimate of the total number of terminals per field, which is, in turn, derived from convergent estimates of the number of granule cells per field. (Estimate 1): there are an estimated 1.92 × 10^6^ granule cells per μl [77]. The number of fields which fit in that volume is (1000/150)^2^ × (1000/200) = ~222, assuming a field depth of 150 μm [20]. The number of granule cells in a field is, therefore 1,920,000/222 = 8649. (Estimate 2): an estimated 350,000 parallel fibres pass through a single Purkinje cell’s territory [77]. Parallel fibres run about 3 mm in each direction from the granule cell soma [94,95]. The Purkinje cell dendritic arbour fills the molecular layer vertically and averages approx. 200 µm wide. Therefore, 6000/150 = 40 fields fit in a row that provides parallel fibres that pass through a Purkinje cell dendritic territory, giving 350,000/40 = 8750 granule cells per field. Estimates vary of the number of granule cells which extend a dendrite into a single glomerulus. Fifty is mid-range. (8750/50) × 4 = 700 glomeruli per field.

Granule cells have 3−5 dendrites, averaging four, and receive input to each of them from a single mossy fibre (so an average granule cell receives four inputs). Estimates of divergence of a single mossy fibre terminal onto granule cells vary [1]. It is unclear how much this reflects biological diversity versus differences in opinion and methods. Here we use a mid-range estimate of 1 : 50 [32,37]. Assuming a mossy fibre terminal receives a single granule cell dendrite from each of *d* granule cells, and given an average of four dendrites per granule cell and *z* granule cells per field, the total number of mossy fibre terminals in a field is *N* = (*z* × 4)/*d* = 700 given z = 8750 and d = 50. A convergent estimate is provided by multiplying the per field number of mossy fibres by the average number of terminals per cluster.

Cluster size varies. We take the range as 4−12 terminals per cluster [20]. We assume the number varies at random and cluster sizes occur with equal probability. A maximum of seven fits in a field (because a field shares the dimensions of the area that encloses a mean cluster). Fields are not anatomically bounded, so many clusters straddle field limits. To reflect that, we assume there is a 1/7 probability that a seven-terminal cluster (or larger) has seven terminals inside a field and also the same probability of any other number. A six-terminal cluster has a 2/7 chance that all six terminals are inside and a 1/7 chance of any other number, and so on.

Table 2 shows the derivation of the expected (i.e. predicted by probability) ratios of terminals contributed to a field by each cluster size (R1), not counting terminals that lie outside when a cluster straddles field boundaries; and the ratios of mossy fibres contributing *n* = 1−7 terminals (R2), regardless of cluster size. R1 ratios are given by the sum of numbers in each row. So, for example, the ratio of cells that end in eight terminals to cells that end in five is 28 : 25. R2 ratios are given by the number of appearances of each number in the grid—so the expected ratio of cells which contribute seven terminals to the number which contribute four is 6 : 12. The ratios effectively give the distributed probability of possible states. For example, there is a probability of 10/(6 + 8 + 10 + 12 + (3 × 9)) = 0.159 that a mossy fibre contributes five terminals to a field (regardless of cluster size). Similarly, there is a probability of ((6 × 28)/((6 × 28) + 27 + 25+22))/7 = 0.099 that a given cell contributes seven terminals to a field.

**Table 2.** Derivation of expected ratios.

size	number of terminals inside							R1
**12**	7	6	5	4	3	2	1	**28**
**11**	7	6	5	4	3	2	1	**28**
**10**	7	6	5	4	3	2	1	**28**
**9**	7	6	5	4	3	2	1	**28**
**8**	7	6	5	4	3	2	1	**28**
**7**	7	6	5	4	3	2	1	**28**
**6**	6	6	5	4	3	2	1	**27**
**5**	5	5	5	4	3	2	1	**25**
**4**	4	4	4	4	3	2	1	**22**
**R2**	6	8	10	12	9	9	9	

If R1 ratios are denoted by a series $x_{4},x_{5}\ldots x_{12},$ and a given element in the series is $x_{j}$, the fraction of terminals provided by a given cluster size is $x_{j}/\left(x_{4}+x_{5}+\ldots +x_{12}\right)$, and this fraction is multiplied by N to give the number of terminals. The number of terminals is divided by the expected mean number of terminals that fall inside field limits (for that cluster size) to give the expected number of mossy fibres that provide them. The number of j-terminal mossy fibres that innervate a field is thus given by

$$N\left[\frac{x_{j}}{\sum_{i=1}^{n}x_{i}}\right]/\left[\frac{x_{j}}{7}\right].$$

The results are shown in table 3. The equal number of mossy fibres (mfs) in each column is what we would expect, because we made it an assumption that all sizes occur with equal probability, and mf values are expected values—i.e. in proportions predicted by their probability. The mf total is the expected number of mossy fibres afferent to a field (which contribute at least one terminal).

**Table 3.** The expected number of mossy fibres which innervate a field.

cluster size	12	11	10	9	8	7	6	5	4	total
**terminals**	81	81	81	81	81	81	78.1	72.3	63.6	700
**mfs**	20.25	20.25	20.25	20.25	20.25	20.25	20.25	20.25	20.25	182.25

R2 ratios—the relative incidence of afferent mossy fibres that contribute seven terminals to a field, six terminals, five and so on, down to one—and the expected number of mossy fibres that contribute them, are shown in table 4.

**Table 4.** The number of mossy fibres which contribute *n* = 1−7 terminals to a field.

*n*	7	6	5	4	3	2	1	total
**R2**	6	8	10	12	9	9	9	63
**R3**	42	48	50	48	27	18	9	
**mfs**	17.34	23.15	28.92	34.72	26.04	26.04	26.04	182.25
**%age**	9.52	12.69	15.87	19.05	14.29	14.29	14.29	

R3 is *n* × R2, giving the ratio of terminals provided by mossy fibres with *n* terminals inside a field. %age is given by (R2/63) × 100. this is the percentage of mossy fibres with *n* terminals inside a field.

From here, the number of mossy fibres that innervate a rank is given by 182.25 × 100 divided by the mean number of clusters per mossy fibre per rank. If a rank receives, on average, five clusters per mossy fibre, approximately 3645 mossy fibres innervate a rank. This is the estimate used in the simulation unless stated otherwise.

*Assumptions*. Cluster sizes occur with equal probability. The probabilities of each outcome when a cluster straddles field boundaries (how many terminals are inside and how many outside) are as stated. A single mossy fibre ends in an average of five terminal clusters per rank.

### A.3. Mossy fibre input rates to a field

Terminal clustering means a field receives multiple copies of most signals. The proportions of mossy fibres afferent to a simulated field that contribute each possible number of terminals (1–7) are generated in simulated steps. In the first step, the simulation randomly generates, for each field, the cluster size ratio. This means, the relative proportions of afferent mossy fibres (which contribute at least one terminal to a field) that end in four terminals, five, six and so on up to 12, assuming they occur with equal probability, disregarding how many terminals fall inside and outside field boundaries. In step two, the ratio of cluster sizes is weighted to reflect the greater probability that large clusters contribute more terminals to a field. In step 3, ratios are converted to the fraction of terminals contributed by each cluster size. When multiplied by the total number of terminals, this gives the number of terminals contributed by each cluster size. In step four, the values obtained in step three are divided up between cells that contribute a single terminal, two terminals, three and so on, to reflect straddling of field limits. The proportions give the number of mossy fibres afferent to a simulated field that contribute each number of terminals up to seven, the maximum.

There is no field-level effect of mossy fibre terminal branching. Terminal clusters arising from the same mossy fibre are separated by a minimum physiological distance, so that a field cannot contain terminals of more than one cluster per mossy fibre. Fields receive a randomly variable number of copies of most signals in the cerebellum and the simulation. In the simulation, copies are added after sampling of mossy fibre rates to obtain rates received by a field. An alternative order would be to add copies to the sampled population and then sample that. We follow anatomy: the number of *rates* received by a field is equal to the number of afferent mossy fibres; the number of *signals* contains duplicate rates.

## Appendix B. Regulation of granule cells by feedback inhibition

It is a long-standing idea [47] that granule cell activity may be regulated by feedback via parallel fibres, which contact Golgi cell apical dendrites. We have previously proposed that gap junctions between apical dendrites may provide the mechanism for Golgi cells to detect the density of parallel fibre activity [96]. Historically, the feedback pathway—mossy fibre → granule cell → Golgi cell → granule cell—has been regarded as a more intuitive candidate for a regulatory role. It was at one time thought that feedforward inhibition might be involved, instead, in control or adjustment of spike timing. We do not incorporate the gap junction model for these reasons: (i) it has appeared previously; (ii) by our calculation, as presented here, the feedforward pathway is the dominant regulator, suggesting the feedback pathway has a supporting, fine-tuning role, so that omitting it has a modest effect on the results; (iii) we might expect that feedforward inhibition is the dominant partner because it operates at local (field) level. Strong local regulation is necessary to ensure that active granule cells are uniformly distributed in the general population; (iv) our scope is a single rank. Parallel fibres have a range of around 3 mm in each direction from the granule cell soma [94,95], so that it would be necessary to model a larger region (about 40 times the size), which extends into the molecular layer, to generate parallel fibre input to a strip of Golgi cells; (v) it is a different (and independent, although related) mechanism; and (vi) they have (related but) different functions. The feed-forward pathway is a local regulator of the fraction of active granule cells (with an effect that flows through to parallel fibres), whereas the feedback pathway is a regulator of the fraction of active parallel fibres only. While the feedback mechanism works by modulating inhibition of granule cells, modulation is very weakly related to local conditions.

## References

[1] Ritzau-Jost A, Delvendahl I, Rings A, Byczkowicz N, Harada H, Shigemoto R, Hirrlinger J, Eilers J, Hallermann S. 2014 Ultrafast action potentials mediate kilohertz signaling at a central synapse. Neuron **84**, 152–163. (10.1016/j.neuron.2014.08.036) 25220814

[2] Jelitai M, Puggioni P, Ishikawa T, Rinaldi A, Duguid I. 2016 Dendritic excitation–inhibition balance shapes cerebellar output during motor behaviour. Nat. Commun. **7**, 13722. (10.1038/ncomms13722) 27976716 PMC5172235

[3] Duguid I, Branco T, Chadderton P, Arlt C, Powell K, Häusser M. 2015 Control of cerebellar granule cell output by sensory-evoked golgi cell inhibition. Proc. Natl Acad. Sci. USA **112**, 13099–13104. (10.1073/pnas.1510249112) 26432880 PMC4620892

[4] Duguid I, Branco T, London M, Chadderton P, Häusser M. 2012 Tonic inhibition enhances fidelity of sensory information transmission in the cerebellar cortex. J. Neurosci. **32**, 11132–11143. (10.1523/jneurosci.0460-12.2012) 22875944 PMC6363100

[5] Walter JT, Khodakhah K. 2006 The linear computational algorithm of cerebellar Purkinje cells. J. Neurosci. **26**, 12861–12872. (10.1523/JNEUROSCI.4507-05.2006) 17167077 PMC6674952

[6] Walter and JT, Khodakhah K. 2009 The advantages of linear information processing for cerebellar computation. Proc. Natl Acad. Sci. USA **106**, p. (10.1073/pnas.0812348106) PMC2657437 19234116

[7] Park S-M, Tara E, Khodakhah K. 2012 Efficient generation of reciprocal signals by inhibition. J. Neurophysiol. **107**, 2453–2462. (10.1152/jn.00083.2012) 22298833 PMC3362251

[8] Delvendahl I, Hallermann S. 2016 The cerebellar mossy fiber synapse as a model for high-frequency transmission in the mammalian CNS. Trends Neurosci. **39**, 722–737. (10.1016/j.tins.2016.09.006) 27771145

[9] Saviane C, Silver RA. 2006 Fast vesicle reloading and a large pool sustain high bandwidth transmission at a central synapse. Nature **439**, 983–987. (10.1038/nature04509) 16496000

[10] Cesana E *et al*. 2010 Novel granule cell-Golgi cell excitatory input in the cerebellar granular layer. In FENS abstr.

[11] Moore JW, Blazis DEJ. 1989 Simulation of a classically conditioned response: a cerebellar neural network implementation of the Sutton–Barto–Desmond model. In Neural models of plasticity (eds JH Byrne, WO Berry), pp. 187–207. Academic Press. (10.1016/b978-0-12-148956-4.50015-2)

[12] Sargent PB, Saviane C, Nielsen TA, DiGregorio DA, Silver RA. 2005 Rapid vesicular release, quantal variability, and spillover contribute to the precision and reliability of transmission at a glomerular synapse. J. Neurosci. **25**, J. (10.1523/JNEUROSCI.2051-05.2005) PMC6725539 16148225

[13] Turecek J, Jackman SL, Regehr WG. 2016 Synaptic specializations support frequency-independent Purkinje cell output from the cerebellar cortex. Cell Rep. **17**, p. (10.1016/j.celrep.2016.11.081) PMC5870134 28009294

[14] Turecek J, Jackman SL, Regehr WG. 2017 Synaptotagmin 7 confers frequency invariance onto specialized depressing synapses. Nature **551**, 503–506. (10.1038/nature24474) 29088700 PMC5892411

[15] Raymond JL, Medina JF. 2018 Computational principles of supervised learning in the cerebellum. Annu. Rev. Neurosci. **41**, 233–253. (10.1146/annurev-neuro-080317-061948) 29986160 PMC6056176

[16] Alviña K, Walter JT, Kohn A, Ellis-Davies G, Khodakhah K. 2008 Questioning the role of rebound firing in the cerebellum. Nat. Neurosci. **11**, 1256–1258. (10.1038/nn.2195) 18820695 PMC2691662

[17] Schonewille M *et al*. 2006 Purkinje cells in awake behaving animals operate at the upstate membrane potential. Nat. Neurosci. **9**, 459–461. (10.1038/nn0406-459) 16568098

[18] Oscarsson O. 1979 Functional units of the cerebellum - sagittal zones and microzones. Trends Neurosci. **2**, 143–145. (10.1016/0166-2236(79)90057-2)

[19] Apps R, Hawkes R. 2009 Cerebellar cortical organization: a one-map hypothesis. Nat. Rev. Neurosci. **10**, 670–681. (10.1038/nrn2698) 19693030

[20] Sultan F, Heck D. 2003 Detection of sequences in the cerebellar cortex: numerical estimate of the possible number of tidal-wave inducing sequences represented. J. Physiol. Paris **97**, 591–600. (10.1016/j.jphysparis.2004.01.016) 15242668

[21] Wu HS, Sugihara I, Shinoda Y. 1999 Projection patterns of single mossy fibers originating from the lateral reticular nucleus in the rat cerebellar cortex and nuclei. J. Comp. Neurol. **411**, 97–118. (10.1002/(sici)1096-9861(19990816)411:13.0.co;2-o) 10404110

[22] Shinoda Y, Sugihara I. 2013 Axonal trajectories of single climbing and mossy fiber neurons in the cerebellar cortex and nucleus. In Handbook of the cerebellum and cerebellar disorders (eds M Manto, F Rossi, DL Gruol, N Koibuchi), pp. 437–467. Dordrecht, The Netherlands: Springer. (10.1007/978-94-007-1333-8_20)

[23] Shinoda Y, Sugihara I, Wu HS, Sugiuchi Y. 2000 The entire trajectory of single climbing and mossy fibers in the cerebellar nuclei and cortex. Prog. Brain Res. **124**, 173–186. (10.1016/S0079-6123(00)24015-6) 10943124

[24] Garwicz M, Ekerot CF, Jorntell H. 1998 Organizational principles of cerebellar neuronal circuitry. News Physiol. Sci **13**, 26–32. 11390755 10.1152/physiologyonline.1998.13.1.26

[25] Ozden I, Sullivan MR, Lee HM, Wang SSH. 2009 Reliable coding emerges from coactivation of climbing fibers in microbands of cerebellar Purkinje neurons. J. Neurosci. **29**, 10463–10473. (10.1523/jneurosci.0967-09.2009) 19710300 PMC2783593

[26] Dean P, Porrill J, Ekerot CF, Jörntell H. 2010 The cerebellar microcircuit as an adaptive filter: experimental and computational evidence. Nat. Rev. Neurosci. **11**, 30–43. (10.1038/nrn2756) 19997115

[27] Eccles JC, Ito M, Szentágothai J. 1967 The cerebellum as a neuronal machine., p. 335. Berlin, Germany: Springer-Verlag.

[28] Barmack NH, Yakhnitsa V. 2008 Functions of interneurons in mouse cerebellum. J. Neurosci. **28**, 1140–1152. (10.1523/jneurosci.3942-07.2008) 18234892 PMC6671404

[29] Hámori J, Szentágothai J. 1966 Participation of Golgi neuron processes in the cerebellar glomeruli: an electron microscope study. Exp. Brain Res. **2**, 35–48. (10.1007/BF00234359) 5921132

[30] D’Angelo E, Solinas S, Mapelli J, Gandolfi D, Mapelli L, Prestori F. 2013 The cerebellar Golgi cell and spatiotemporal organization of granular layer activity. Front. Neural Circuits **7**, 93. (10.3389/fncir.2013.00093) 23730271 PMC3656346

[31] Rossi DJ, Hamann M. 1998 Spillover-mediated transmission at inhibitory synapses promoted by high affinity alpha6 subunit GABA(A) receptors and glomerular geometry. Neuron **20**, 783–795. (10.1016/s0896-6273(00)81016-8) 9581769

[32] Jakab RL, Hámori J. 1988 Quantitative morphology and synaptology of cerebellar glomeruli in the rat. Anat. Embryol. **179**, 81–88. (10.1007/BF00305102) 3213958

[33] Nusser Z, Sieghart W, Somogyi P. 1998 Segregation of different GABAA receptors to synaptic and extrasynaptic membranes of cerebellar granule cells. J. Neurosci. **18**, 1693–1703. (10.1523/JNEUROSCI.18-05-01693.1998) 9464994 PMC6792611

[34] Brickley SG, Cull-Candy SG, Farrant M. 1996 Development of a tonic form of synaptic inhibition in rat cerebellar granule cells resulting from persistent activation of GABAA receptors. J. Physiol. **497**, 753–759. (10.1113/jphysiol.1996.sp021806) 9003560 PMC1160971

[35] Kanichay RT, Silver RA. 2008 Synaptic and cellular properties of the feedforward inhibitory circuit within the input layer of the cerebellar cortex. J. Neurosci. **28**, 8955–8967. (10.1523/jneurosci.5469-07.2008) 18768689 PMC2923072

[36] Dieudonné S. 1998 Submillisecond kinetics and low efficacy of parallel fibre‐Golgi cell synaptic currents in the rat cerebellum. J. Physiol. **510**, 845–866. (10.1111/j.1469-7793.1998.845bj.x) 9660898 PMC2231065

[37] Gao Z, Proietti-Onori M, Lin Z, ten Brinke MM, Boele HJ, Potters JW, Ruigrok TJH, Hoebeek FE, De Zeeuw CI. 2016 Excitatory cerebellar nucleocortical circuit provides internal amplification during associative conditioning. Neuron **89**, 645–657. (10.1016/j.neuron.2016.01.008) 26844836 PMC4742536

[38] Mitchell SJ, Silver RA. 2000 GABA spillover from single inhibitory axons suppresses low-frequency excitatory transmission at the cerebellar glomerulus. J. Neurosci. **20**, 8651–8658. (10.1523/jneurosci.20-23-08651.2000) 11102470 PMC6773066

[39] Mitchell SJ, Silver RA. 2000 Glutamate spillover suppresses inhibition by activating presynaptic mGluRs. Nature **404**, 498–502. (10.1038/35006649) 10761918

[40] Rancz EA, Ishikawa T, Duguid I, Chadderton P, Mahon S, Häusser M. 2007 High-fidelity transmission of sensory information by single cerebellar mossy fibre boutons. Nature **450**, 1245–1248. (10.1038/nature05995) 18097412 PMC5881887

[41] Chadderton P, Margrie TW, Häusser M. 2004 Integration of quanta in cerebellar granule cells during sensory processing. Nature **428**, 856–860. (10.1038/nature02442) 15103377

[42] Bengtsson F, Jörntell H. 2009 Sensory transmission in cerebellar granule cells relies on similarly coded mossy fiber inputs. Proc. Natl Acad. Sci. USA **106**, 2389–2394. (10.1073/pnas.0808428106) 19164536 PMC2650166

[43] Billings G, Piasini E, Lőrincz A, Nusser Z, Silver RA. 2014 Network structure within the cerebellar input layer enables lossless sparse encoding. Neuron **83**, 960–974. (10.1016/j.neuron.2014.07.020) 25123311 PMC4148198

[44] Jörntell H, Ekerot CF. 2006 Properties of somatosensory synaptic integration in cerebellar granule cells in vivo. J. Neurosci. **26**, 11786–11797. (10.1523/jneurosci.2939-06.2006) 17093099 PMC6674774

[45] Gilbert M, Chris Miall R. 2022 How and why the cerebellum recodes input signals: an alternative to machine learning. Neuroscience **28**, 206–221. (10.1177/1073858420986795) PMC9136479 33559532

[46] Cesana E, Pietrajtis K, Bidoret C, Isope P, D’Angelo E, Dieudonne S, Forti L. 2013 Granule cell ascending axon excitatory synapses onto Golgi cells implement a potent feedback circuit in the cerebellar granular layer. J. Neurosci. **33**, 12430–12446. (10.1523/jneurosci.4897-11.2013) 23884948 PMC6618671

[47] Albus JS. 1971 A theory of cerebellar function. Math. Biosci. **10**, 25–61. (10.1016/0025-5564(71)90051-4)

[48] Hámori J. 1992 Anatomy and neurochemical anatomy of the cerebellum. In Foundations of neurology cerebellar degenerations: clinical neurobiology (ed. A Plaitakis), pp. 11–57. Kluwer Academic. (10.1007/978-1-4615-3510-2_2)

[49] Palay SL, Chan-Palay V. 1974 Cerebellar cortex: cytology and organization. Berlin, Germany: Springer.

[50] Lange W. 1974 Regional differences in the distribution of Golgi cells in the cerebellar cortex of man and some other mammals. Cell Tissue Res. **153**. (10.1007/bf00226610) 4442086

[51] Miles FA, Fuller JH, Braitman DJ, Dow BM. 1980 Long-term adaptive changes in primate vestibuloocular reflex. III. Electrophysiological observations in flocculus of normal monkeys. J. Neurophysiol. **43**, 1437–1476. (10.1152/jn.1980.43.5.1437) 6768853

[52] Edgley SA, Lidierth M. 1987 The discharges of cerebellar Golgi cells during locomotion in the cat. J. Physiol. **392**, p. (10.1113/jphysiol.1987.sp016782) PMC1192306 3446782

[53] Galliano E, Mazzarello P, D’Angelo E. 2010 Discovery and rediscoveries of Golgi cells. J. Physiol. **588**, p. (10.1113/jphysiol.2010.189605) PMC2998217 20581044

[54] Eccles JC, Llinás R, Sasaki K. 1964 Golgi cell inhibition in the cerebellar cortex. Nature **204**, 1265–1266. (10.1038/2041265a0) 14254404

[55] Holtzman T, Rajapaksa T, Mostofi A, Edgley SA. 2006 Different responses of rat cerebellar Purkinje cells and Golgi cells evoked by widespread convergent sensory inputs. J. Physiol **574**, 491–507. (10.1113/jphysiol.2006.108282) 16709640 PMC1817778

[56] Vos BP, Volny‐Luraghi A, De Schutter E. 1999 Cerebellar Golgi cells in the rat: receptive fields and timing of responses to facial stimulation. Eur. J. Neurosci. **11**, 2621–2634. (10.1046/j.1460-9568.1999.00678.x) 10457161

[57] Hartmann MJ, Bower JM. 2001 Tactile responses in the granule cell layer of cerebellar folium crus iia of freely behaving rats. J. Neurosci. **21**, 3549–3563. (10.1523/jneurosci.21-10-03549.2001) 11331384 PMC6762470

[58] Arenz A, Silver RA, Schaefer AT, Margrie TW. 2008 The contribution of single synapses to sensory representation in vivo. Science **321**, 977–980. (10.1126/science.1158391) 18703744 PMC2771362

[59] van Kan PLE, Gibson AR, Houk JC. 1993 Movement-related inputs to intermediate cerebellum of the monkey. J. Neurophysiol. **69**, 1–1. (10.1152/jn.1993.69.3.1-a) 8433135

[60] Giovannucci A *et al*. 2017 Cerebellar granule cells acquire a widespread predictive feedback signal during motor learning. Nat. Neurosci. **20**, 727–734. (10.1038/nn.4531) 28319608 PMC5704905

[61] Pietrajtis K, Dieudonné S. 2013 Golgi neurons. In Handbook of the cerebellum and cerebellar disorders (eds M Manto, JD Schmahmann, F Rossi, DL Gruol, N Koibuchi), pp. 829–852. Dordrecht, The Netherlands: Springer Netherlands. (10.1007/978-94-007-1333-8_34)

[62] Eyre MD, Nusser Z. 2016 Only a minority of the inhibitory inputs to cerebellar Golgi cells originates from local GABAergic cells. Eneuro **3**, 2. (10.1523/eneuro.0055-16.2016) PMC4876488 27257627

[63] Dumoulin A, Triller A, Dieudonné S. 2001 IPSC kinetics at identified gabaergic and mixed GABAergic and glycinergic synapses onto cerebellar Golgi cells. J. Neurosci. **21**, 6045–6057. (10.1523/jneurosci.21-16-06045.2001) 11487628 PMC6763194

[64] Hull C, Regehr WG. 2012 Identification of an inhibitory circuit that regulates cerebellar Golgi cell activity. Neuron **73**, 149–158. (10.1016/j.neuron.2011.10.030) 22243753 PMC3259536

[65] Vervaeke K, Lőrincz A, Gleeson P, Farinella M, Nusser Z, Silver RA. 2010 Rapid desynchronization of an electrically coupled interneuron network with sparse excitatory synaptic input. Neuron **67**, 435–451. (10.1016/j.neuron.2010.06.028) 20696381 PMC2954316

[66] Roš H, Sachdev RNS, Yu Y, Šestan N, McCormick DA. 2009 Neocortical networks entrain neuronal circuits in cerebellar cortex. J. Neurosci. **29**, 10309–10320. (10.1523/jneurosci.2327-09.2009) 19692605 PMC3137973

[67] Ruigrok TJH, Hensbroek RA, Simpson JI. 2011 Spontaneous activity signatures of morphologically identified interneurons in the vestibulocerebellum. J. Neurosci. **31**, 712–724. (10.1523/jneurosci.1959-10.2011) 21228180 PMC6623423

[68] Rasmussen A, Zucca R, Jirenhed DA, Johansson F, Ortenblad C, Svensson P, Hesslow G. 2014 Golgi cell activity during eyeblink conditioning in decerebrate ferrets. Cerebellum **13**, 42–45. (10.1007/s12311-013-0518-3) 23982588

[69] DiGregorio DA, Rothman JS, Nielsen TA, Silver RA. 2007 Desensitization properties of AMPA receptors at the cerebellar mossy fiber–granule cell synapse. J. Neurosci. **27**, 8344–8357. (10.1523/jneurosci.2399-07.2007) 17670981 PMC6147216

[70] DiGregorio DA, Nusser Z, Silver RA. 2002 Spillover of glutamate onto synaptic AMPA receptors enhances fast transmission at a cerebellar synapse. Neuron **35**, 521–533. (10.1016/s0896-6273(02)00787-0) 12165473

[71] Mapelli L, Solinas S, D’Angelo E. 2014 Integration and regulation of glomerular inhibition in the cerebellar granular layer circuit. Front. Cell. Neurosci. **8**, 55. (10.3389/fncel.2014.00055) 24616663 PMC3933946

[72] Chabrol FP, Arenz A, Wiechert MT, Margrie TW, DiGregorio DA. 2015 Synaptic diversity enables temporal coding of coincident multisensory inputs in single neurons. Nat. Neurosci. **18**, 718–727. (10.1038/nn.3974) 25821914 PMC4413433

[73] Sherman SM, Guillery RW. 2011 Distinct functions for direct and transthalamic corticocortical connections. J. Neurophysiol. **106**, 1068–1077. (10.1152/jn.00429.2011) 21676936

[74] Rossi DJ, Hamann M, Attwell D. 2003 Multiple modes of GABAergic inhibition of rat cerebellar granule cells. J. Physiol. **548**, 97–110. (10.1111/j.1469-7793.2003.00097.x) 12588900 PMC2342786

[75] Hansel C, Linden DJ, D’Angelo E. 2001 Beyond parallel fiber LTD: the diversity of synaptic and non-synaptic plasticity in the cerebellum. Nat. Neurosci. **4**, 467–475. (10.1038/87419) 11319554

[76] Gao Z, van Beugen BJ, De Zeeuw CI. 2012 Distributed synergistic plasticity and cerebellar learning. Nat. Rev. Neurosci. **13**, 619–635. (10.1038/nrn3312) 22895474

[77] Harvey RJ, Napper RMA. 1988 Quantitative study of granule and Purkinje cells in the cerebellar cortex of the rat. J. Comp. Neurol. **274**, 151–157. (10.1002/cne.902740202) 3209738

[78] Harvey RJ, Napper RM. 1991 Quantitative studies on the mammalian cerebellum. Prog. Neurobiol. **36**, 437–463. (10.1016/0301-0082(91)90012-p) 1947173

[79] Napper RMA, Harvey RJ. 1988 Number of parallel fiber synapses on an individual Purkinje cell in the cerebellum of the rat. J. Comp. Neurol. **274**, 168–177. (10.1002/cne.902740204) 3209740

[80] Napper RMA, Harvey RJ. 1988 Quantitative study of the Purkinje cell dendritic spines in the rat cerebellum. J. Comp. Neurol. **274**, 158–167. (10.1002/cne.902740203) 3209739

[81] Isope P, Barbour B. 2002 Properties of unitary granule cell→Purkinje cell synapses in adult rat cerebellar slices. J. Neurosci. **22**, 9668–9678. (10.1523/jneurosci.22-22-09668.2002) 12427822 PMC6757845

[82] Ekerot C, Jörntell H. 2001 Parallel fibre receptive fields of Purkinje cells and interneurons are climbing fibre‐specific. Eur. J. Neurosci. **13**, 1303–1310. (10.1046/j.0953-816x.2001.01499.x) 11298790

[83] Dugué GP, Tihy M, Gourévitch B, Léna C. 2017 Cerebellar re-encoding of self-generated head movements. eLife **6**. (10.7554/elife.26179) PMC5489315 28608779

[84] Chen S, Augustine GJ, Chadderton P. 2016 The cerebellum linearly encodes whisker position during voluntary movement. eLife **5**, e10509. (10.7554/elife.10509) 26780828 PMC4737656

[85] Herzfeld DJ, Kojima Y, Soetedjo R, Shadmehr R. 2015 Encoding of action by the Purkinje cells of the cerebellum. Nature **526**, 439–442. (10.1038/nature15693) 26469054 PMC4859153

[86] Hong S, Negrello M, Junker M, Smilgin A, Thier P, De Schutter E. 2016 Multiplexed coding by cerebellar Purkinje neurons. eLife **5**. (10.7554/elife.13810) PMC4961467 27458803

[87] Fujita M. 1982 Adaptive filter model of the cerebellum. Biol. Cybern. **45**, 195–206. (10.1007/BF00336192) 7171642

[88] Marr D. 1969 A theory of cerebellar cortex. J. Physiol **202**, 437–470. (10.1113/jphysiol.1969.sp008820) 5784296 PMC1351491

[89] Apps R, Garwicz M. 2005 Anatomical and physiological foundations of cerebellar information processing. Nat. Rev. Neurosci. **6**, 297–311. (10.1038/nrn1646) 15803161

[90] Ji Z, Hawkes R. 1994 Topography of Purkinje cell compartments and mossy fiber terminal fields in lobules ii and iii of the rat cerebellar cortex: spinocerebellar and cuneocerebellar projections. Neuroscience **61**, 935–954. (10.1016/0306-4522(94)90414-6) 7530818

[91] Larsell O. 1967 The comparative anatomy and histology of the cerebellum from myxinoids through birds. Minneapolis, MN: The University of Minnesota Press.

[92] Larsell O. 1970 The comparative anatomy and histology of the cerebellum from monotremes through apes. Minneapolis, MN: The University of Minnesota Press.

[93] Gilbert M. 2024 Research software supporting the publication ’What if...? Computational anatomy in the granular layer of the cerebellar cortex'. See 10.25500/edata.bham.00001160.

[94] Mugnaini E. 1983 The length of cerebellar parallel fibers in chicken and rhesus monkey. J. Comp. Neurol. **220**, 7–15. (10.1002/cne.902200103) 6643718

[95] Brand S, Dahl AL, Mugnaini E. 1976 The length of parallel fibers in the cat cerebellar cortex. An experimental light and electron microscopic study. Exp. Brain Res. **26**. (10.1007/bf00235248) 61126

[96] Gilbert M, Rasmussen A. 2024 Gap junctions may have a computational function in the cerebellum: a hypothesis. Cerebellum **23**, 1903–1915. (10.1007/s12311-024-01680-3) 38499814 PMC11489243

[97] Gilbert M, Rasmussen A. 2025 Supplementary material from: The cerebellum converts input data into a hyper low-resolution granule cell code with spatial dimensions: a hypothesis. Figshare. (10.6084/m9.figshare.c.7676032) PMC11937928 40144291

